# Norway spruce at the trailing edge: the effect of landscape configuration and composition on climate resilience

**DOI:** 10.1007/s10980-019-00964-y

**Published:** 2020-01-11

**Authors:** Juha Honkaniemi, Werner Rammer, Rupert Seidl

**Affiliations:** Institute of Silviculture, University of Natural Resources and Life Sciences (BOKU), Peter-Jordan Strasse 82, 1190 Vienna, Austria

**Keywords:** Landscape management, Natural disturbance, Norway spruce, Resilience, Spatial configuration, Species composition

## Abstract

**Context:**

Norway spruce (*Picea abies*) is one of the most widespread tree species in Europe’s forests. Due to its high economic value it has been strongly favored by management, especially at the trailing edge of its natural distribution. However, disturbances from wind and bark beetles are increasingly impacting these forests, and their resilience under climate change has been called into question recently.

**Objectives:**

We quantified the effects of landscape configuration and composition on (1) the risk from natural disturbances, and (2) on the overall resilience of Norway spruce to changing climate at the trailing edge.

**Methods:**

We simulated the dynamics of a 9183 ha forest landscape in Eastern Austria over 190 years. We used the simulation model iLand to experimentally study a wide range of landscape compositions and configurations under five different climate scenarios.

**Results:**

Natural disturbances increased considerably under all future climate scenarios. Dispersing Norway spruce throughout the landscape in mixed stands resulted in the highest levels of climate resilience. Reducing the percentage of Norway spruce on the landscape increased the resilience of the remaining Norway spruce trees, yet landscape configuration generally had a stronger effect on resilience than composition.

**Conclusions:**

The resilience of Norway spruce at the trailing edge of its distribution is challenged by climate change, and considerable efforts are needed to sustain these ecosystems. While currently discussed adaptation measures focus largely on the stand level, we show that modifying landscape composition and configuration can be used to foster Norway spruce resilience while maintaining socio-economically relevant proportions of Norway spruce.

**Electronic supplementary material:**

The online version of this article (doi:10.1007/s10980-019-00964-y) contains supplementary material, which is available to authorized users.

## Introduction

**Fig. 1:**
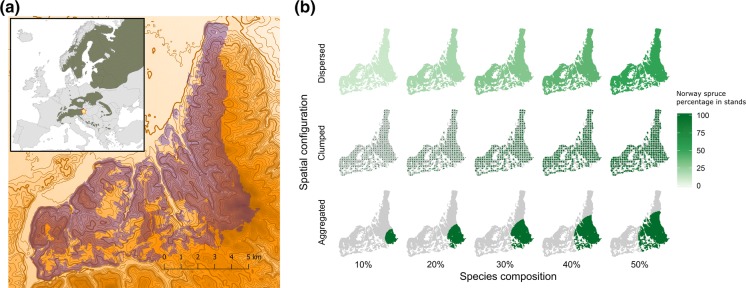
**a** The Bucklige Welt study landscape in Eastern Austria, indicating its elevation gradient (contours with 20 m interval (thicker line every 100 meters) and color from light (low elevation 200 m) to dark (high elevation 750 m) brown and forested area for simulations (blue). The inset map of Europe shows the location of the landscape at the trailing edge of Norway spruces range distribution (indicated in dark green, de Vries et al. 2015). **b** Visualization of the 15 simulated landscape composition and configuration scenarios

Norway spruce (*Picea abies* [L.] Karst) is one of the most iconic tree species of the forest ecosystems of Eurasia. Its natural distribution ranges from Siberia to Fennoscandia through the Baltic countries all the way to the mountain ranges of Central Europe (de Vries et al. 2015) (Fig. 1a). Norway spruce prefers cool and wet climate, fertile soils and—being a relatively shade tolerant species—grows well in mixtures with other tree species. Economically, it is currently one of the most important tree species in Europe. Its valuable timber and relative ease of management have resulted in a considerable human-induced increase in Norway spruce in many areas (Johann et al. 2004). As a result, there are 5.7–7.3 M ha of pure Norway spruce forest at the margins of or even outside of the species’ natural range in Europe today (von Teuffel et al. 2004).

Climate change is increasingly challenging Norway spruce throughout its range (Schlyter et al. 2006; Hanewinkel et al. 2013). A relatively shallow root system makes the species prone to drought stress (Pretzsch et al. 2013; Zang et al. 2014) and increases the risk for wind damage (Peltola et al. 1999; Seidl et al. 2014a). In addition, past decades have shown an increasing vulnerability of Norway spruce to European spruce bark beetle (*Ips typographus* L., Coleoptera: Curculionidae) outbreaks. These outbreaks are frequently triggered by major storm events and severe drought (Lausch et al. 2013; Stadelmann et al. 2014; Seidl et al. 2016c). Disturbances are expected to increase in the future particularly at the warm and dry edge of the current distribution of Norway spruce, as climate change progresses over the coming decades (Jacob et al. 2014). Consequently, the resilience of Norway spruce at the trailing edge of its distribution (i.e. the warm/ dry distributional margins which are increasingly under pressure in a warming world) has been called into question lately (Hlásny et al. 2017; Seidl et al. 2017b).

Resilience is a powerful concept for assessing the viability of a species under changing conditions. The concept of resilience has been applied in numerous ways in ecology (Brand and Jax 2007), with one being a balance between the impact of a perturbation (e.g., climate change) and the vegetation recovery to the pre-perturbation state after it (Carpenter et al. 2001; Ingrisch and Bahn 2018). This particular rendering of resilience can be especially insightful for studying the margins of a species range, where impacts that exceed the recovery capacity of the system indicate reduced viability and eventually range contraction. While the range margins of a species are influenced by a variety of factors (e.g. human impact, phenotypic plasticity) (Sagarin et al. 2006), environmental conditions are often sub-optimal (Sexton et al. 2009), making them particularly prone to changing environmental conditions. In the specific context of Norway spruce, previous efforts have mainly focused on reducing the impacts of climate change via two approaches. Firstly, thinning interventions reducing stem density and increasing the availability of scarce resources (such as water) for the remaining trees have been shown to reduce the vulnerability of Norway spruce to drought and foster recovery after drought (Laurent et al. 2003; Kohler et al. 2010). Secondly, mixing Norway spruce with other species can reduce mortality from wind and beetles, and increase stand stability (Valinger and Fridman 2011; Jactel et al. 2017). While these approaches focus on the tree- to stand-level, landscape-level approaches to fostering Norway spruce resilience to climate change remain largely untested to date.

Landscape structure can make an important contribution to the resilience of ecosystems (Cumming 2011). Landscape structure can be characterized as the composition (e.g., the percentage of Norway spruce on the overall tree species composition) and configuration (i.e., spatial characteristics like the shape and connectivity of patches of a given species) of a landscape. The composition and configuration of landscapes are particularly relevant when considering host-specific and spatially-contagious processes, such as the spread of insect outbreaks (Johnson et al. 2004; Seidl et al. 2016b). Furthermore, the fact that historic land-use has substantially altered landscape structure compared to natural ecosystems underlines that forest management can actively modulate forest ecosystems at the landscape scale (Munteanu et al. 2015; Bebi et al. 2017). Studies of forest landscape structure and its effect in the context of climate resilience remain rare, however, as (i) landscape composition and configuration are rarely independent, making observational studies challenging, and (ii) manipulative studies at the landscape scale are resource intensive and suffer from limited comparability. Landscape simulation models are promising tools in this regard (Shifley et al. 2017), as they enable landscape-scale experiments (including replication) while granting consistency in environmental factors.

Using a forest landscape model, we studied the resilience of Norway spruce at the margins of its geographic distribution. Our objectives were to quantify the effects of landscape configuration and composition on (1) the future risk from natural disturbances, namely wind and bark beetle outbreaks (as a key process contributing to the climate sensitivity of Norway spruce), and subsequently (2) on the overall resilience of Norway spruce to changing climate at its warm and dry range edge. We focused on a forest landscape in the lowlands of Eastern Austria, due to its representativeness for Norway spruce forests at the trailing edge. For our study landscape we tested the hypothesis that configurations that reduce spatial connectivity of Norway spruce on the landscape reduce the risk of natural disturbances (Zeng et al. 2010; Seidl et al. 2016b). In addition, based on the strong evidence of positive effects of tree species mixing at the stand level (Bauhus et al. 2017; Jactel et al. 2018), we hypothesized that landscape configuration (i.e., whether tree species are planted in mixed stands or mono-specific stands) is more important for the overall resilience of Norway spruce than landscape composition (i.e., the percentage of each species at the landscape scale).

## Materials and methods

### Study area

Our study landscape is located in the lowlands of Eastern Austria (47.70 N, 16.25 E) at the trailing edge of Norway spruces natural distribution (Fig. 1a). The landscape covers a total area of 9183 ha with a stockable forest area of 6700 ha. The elevation range of the landscape extends from 270 to 735 meters a.s.l. and the climate is characterized as warm, subcontinental Pannonic (Sundseth 2009). The long term average (1981–2010) mean annual temperature varies from 7.9 to 9.6 °C (decreasing with elevation) and the annual precipitation ranges from 640 to 940 mm (increasing with elevation). The soils are predominately cambisols on crystalline bedrock. The school forest of the University of Natural Resources and Life Sciences Vienna (BOKU) is situated within the landscape, covering a forest area of 1135 ha (Supplementary material S1).

The potential natural vegetation (PNV) of the landscape is dominated by European beech (*Fagus sylvatica*) and, to a lesser degree, by Silver fir (*Abies alba* Mill.). Norway spruce would naturally occur only in low percentages (below 5%). However, due to intensive past forest management the current percentage of Norway spruce on the overall growing stock of the landscape is 45%, with Norway spruce frequently being planted in pure stands in the past. Storm events with subsequent bark beetle outbreaks are the most important natural disturbance agents of Norway spruce in the area (Thom et al. 2013).

### The forest landscape simulation model iLand

We simulated forest and disturbance dynamics in the study landscape using iLand (Seidl et al. 2012) (http://iland.boku.ac.at). iLand is a spatially-explicit, process-based model simulating ecosystem processes from the level of single trees to the landscape scale. The model was specifically designed to study the complex interactions between climate, forest dynamics and natural disturbances. Thus, iLand is particularly suited to study the resilience of forest ecosystem to climate change, and has been successfully applied to questions of resilience recently (Seidl et al. 2014b, 2017b). In the following we focus on describing how important processes influencing forest resilience are modeled in iLand, for a more general description of the model see Seidl et al. (2012) and Thom et al. (2017b). Recovery processes (i.e. the regeneration and growth of trees) are simulated as a function of local forest composition (e.g., seed availability as determined by the spatial distribution of mature trees) and structure (e.g., determining light availability), accounting for important biotic interactions within forest ecosystems. Furthermore, the ability to grow and recover from perturbations is fundamentally influenced by the abiotic environment, specifically weather (here the daily variation in temperature, precipitation, radiation, and vapor pressure deficit) and site conditions (e.g., soil water holding capacity, site fertility). Gross primary productivity is calculated by means of a radiation use efficiency approach (Landsberg and Waring 1997). After accounting for autotrophic respiration the obtained net primary productivity is allocated to different tree compartments using allometric ratios (see more http://iland.boku.ac.at/growth).

Tree mortality is a key process in the context of forest resilience. In this regard iLand accounts for both individual tree mortality (i.e. as the result of stress from carbon starvation) and large scale mortality events due to natural disturbances. The natural disturbance agents of particular relevance in our study system are wind and bark beetles, which are both simulated in a highly detailed process-based manner in iLand (Seidl et al. 2014a; Seidl and Rammer 2017). The wind disturbance module operates at the grain of individual trees. It takes wind speed data as input and simulates storm events dynamically based on a dose–response approach, taking into account changes in stand structure during a wind event. The bark beetle disturbance module focuses on *I. typographus* and simulates beetle phenology and development as well as spatially-explicit dispersal of beetles (Seidl and Rammer 2017). The beetle phenology routine predicts the spring swarming, colonization and brood development as a function of temperature, with different thresholds considered for each process (Baier et al. 2007; Seidl et al. 2007) (see more http://iland.boku.ac.at/bark+beetle+disturbance). The spatially-explicit dispersal is simulated for each beetle cohort (i.e., a group of beetles leaving the tree approximately at the same time) in two stages; first the random flight of beetle cohorts according to a symmetrical dispersal kernel (Kautz et al. 2014, max. dispersal distance 514 m), second the active search of beetles for a suitable host in the local environment (30 m). The interaction of wind and bark beetles is explicitly simulated by increased colonization and reproduction success in wind-disturbed trees. In addition, stress (as indicated by a trees carbon balance) affects host colonization as more beetles are needed to overcome the defense of healthy, vigorous trees (Huang et al. 2019). Forest management is implemented via an agent-based approach, simulating adaptive management regimes dynamically adapting management rules for each simulated stand (Rammer and Seidl 2015). Stand treatment programs are specified as corridors for when and how stands are planted, thinned, and harvested. These generic stand treatment programs are subsequently dynamically adapted to local stand conditions by the management agents, accounting for landscape-scale constraints (e.g., adjacency rules, sustainable harvest levels). Moreover, the iLand management module includes routines for salvage logging after storm events and the use of trap trees as a sanitary measure to prevent bark beetle outbreaks.

iLand has been parameterized, tested and evaluated in several landscapes in Central Europe, with a focus on mountain forest ecosystems (Thom et al. 2017a; Seidl et al. 2018). The current study expands the altitudinal and climatic gradient of landscapes simulated with iLand to the warm lowlands of Central Europe, where the environmental conditions differ substantially from previous iLand applications. Therefore, we carried out a thorough model evaluation for our current study landscape, following the principles of pattern-oriented modelling (Grimm et al. 2005). Specifically, we focused our evaluation on simulated productivity at the tree species level, potential natural vegetation (PNV) dynamics and wind and bark beetle levels (Supplementary material S1).

### Scenarios of landscape configuration and composition

In order to assess the effects of landscape structure, we studied five different levels of Norway spruce percentage in three different spatial configurations, resulting in a total of 15 scenarios of landscape structure (Fig. 1b). The three different spatial configurations of Norway spruce in the landscape considered were (i) *dispersed*, where Norway spruce was evenly distributed across the landscape in mixed stands; (ii) *clumped*, where equally sized groups of monospecific Norway spruce stands were distributed regularly throughout the landscape at a distance of 514 m (i.e. approximately the maximum dispersal range of *I. typographus*, Kautz et al. (2011)) between the centroids of the groups (with the aim to inhibit the spread of bark beetle outbreaks across the landscape), and (iii) *aggregated*, where a single, contiguous monospecific block of Norway spruce, situated in the highest elevations of the landscape, was simulated. These three spatial configuration scenarios were simulated for five different levels of Norway spruce in the landscape, varying Norway spruce percentage between 10–50% (the latter roughly corresponding to current Norway spruce percentages in our study landscape) in 10% increments. The 15 different landscape structures were populated with stand information available from local inventory data (Supplementary material S2). In order to control for structural legacy effects, we assumed all scenarios of landscape structure followed a normal forest distribution, i.e., an even distribution of stand ages across the landscape (range 0–100 years) with an even-aged structure within stands. Model initializations for the specified stands were derived via a legacy spin-up procedure (Thom et al. 2018) (Supplementary material S2). In addition, to control the structural changes during the simulations, we applied a common forest management regime across all simulated stands. Stand treatment programs were designed to mimic current forest management of Norway spruce in our study area (Supplementary material S3).

### Climate scenarios

We simulated the landscape dynamics under five climate scenarios over 190 years (2010–2200) (Table 1). First, we assumed a continuation of historic climate conditions by randomly resampling years from the period 1981–2010 with replacement to derive a baseline for the assessment of climate change resilience. Furthermore, four alternative climate change scenarios derived from different GCM-RCM combinations were considered, spanning a wide range of possible future climate conditions. Specifically, we selected one scenario under RCP 4.5 forcing (“moderate”), and three scenarios under RCP 8.5 forcing (“warm,” “warm and wet,” and “hot and dry”). Climate scenario data were available until 2100, and we derived the climate for the twenty-second century by resampling years from the period 2080 to 2100 in the respective scenario, assuming a stabilization of the climate system. For all climate scenarios, the original climate data obtained at 1 km resolution was downscaled to a grain of 100 m grid cells using kriging (Supplementary material S4).

**Table 1:** The climate scenarios simulated, as characterized by mean annual temperature (*Tmean*) and precipitation sum (*Prec*) for the landscape over the 190 year simulation period

Climate scenario	*Tmean* (°C)	σ_T_ (°C)	*Prec* (mm)	σ_Prec_ (mm)	Number of storm events
Historic (1980–2010)	8.6	7.9–9.6	794	639–943	20
Moderate (EC-EARTH and KNMI-RACMO22E RCP4.5)	10.4	9.6–11.4	835	674–986	20
Warm (EC-EARTH and KNMI-RACMO22E RCP8.5)	11.9	11.1–12.8	812	660–955	12
Warm and wet (IPSL-CM5A-MR and IPSL-INERIS-WRF331F RCP8.5)	11.7	10.9–12.6	933	761–1112	26
Hot and dry (HadGEM2-ES and CLMcom-CCLM4-8-17 RCP8.5)	12.8	12.0–13.8	645	528–771	11

Within-landscape variation of temperature (σ_T_) and precipitation (σ_Prec_) are indicated as the min-max range on the landscape. The total number of storm events was derived from climate model data (with GCM-RCM combinations and representative concentration pathways in parenthesis) using a maximum gust wind speed cutoff of 33.3 m s^−1^

Storm events were derived directly from the wind data available for the climate change scenarios, here defined as days with a maximum gust wind speed (2 s gusts) of above 33.3 m s^−1^. The direction for each storm event was randomly drawn from a distribution of wind directions of the closest weather station (i.e., the city of Wiener Neustadt). National wind atlas (Krenn et al. 2011) data were used to adjust the wind speed according to the topography of the landscape. To account for the high level of stochasticity associated with the occurrence of individual storm events we conducted 20 replicated simulations of each landscape structure scenario, varying the timing of each storm event across replicates. In addition, the initial value for the annual probability of bark beetle occurrence per hectare was varied between replicates (0.0005–0.0025). In total, we simulated 75 scenarios (5 landscape composition scenarios × 3 landscape configuration scenarios × 5 climate scenarios) over 190 years for the landscape with each simulation replicated 20 times.

### Analyses

The main objectives of our study were to analyze the effects of landscape composition (i.e., Norway spruce percentage) and configuration (i.e., spatial distribution of Norway spruce stands in the landscape) on (i) natural disturbance processes, and (ii) Norway spruce resilience to climate change. To address the first objective we dynamically simulated natural disturbances from wind and bark beetles in all 15 scenarios of landscape structure. We used the average annual timber volume affected by disturbances (*DisturbanceVolume*, Eq. 1) to quantify the effects of landscape structure on disturbance.

1 $$DisturbanceVolume_{ij} \; = \;\frac{1}{n}\mathop \sum \limits_{t = 1}^{n} \left( {mortality\; by\; agent\;X,\;m^{3} } \right)_{tij}$$

where *t *= *year, n *= *number of years simulated, i *= *climate change scenario, j *= *landscape structure scenario.*

In addition, given the importance of amplifying interactions for the wind–bark beetle disturbance regime (Stadelmann et al. 2014; Seidl and Rammer 2017), we quantified their interaction and tested how different configurations and compositions modulate disturbance interactions. Specifically, we calculated the 3 year cumulative timber volume disturbed by bark beetles before and after major storm events (occurring at least 6 years apart). We then normalized the wind effect on bark beetle outbreaks to the timber disturbed by wind to obtain a relative measure of interaction strength (Eq. 2).

2 $$Interaction\;Strength = {{\left( {\sum BBvolume_{after} - \sum BBvolume_{before} } \right)} \mathord{\left/ {\vphantom {{\left( {\sum BBvolume_{after} - \sum BBvolume_{before} } \right)} {\text{WindVolume}}}} \right. \kern-0pt} {\text{WindVolume}}}$$

*where BBvolume = cumulative timber volume disturbed by bark beetles, before = 3 years before storm event, after = 3 years after storm event, WindVolume = wind-disturbed volume of the event.*

The second objective was to assess the Norway spruce resilience to climate change in the different scenarios of landscape structure. Resilience has been conceptualized in a wide variety of ways (Brand and Jax 2007). Following Ingrisch and Bahn (2018), we quantified resilience based on a bivariate analysis framework, where the two dimensions are the impact of a perturbation on the ecosystem and the ecosystems ability to recovery from this impact. With regard to impact, a major expected effect of climate change is an increase in tree mortality and failure to recover to previous system states (Allen et al. 2015; Millar and Stephenson 2015; Seidl et al. 2017a). We thus quantified the impact of climate change on Norway spruce as the mean annual mortality rate (m^3^ year^−1^) of the species in the different climate scenarios (Eq. 3).

3 $$Impact_{ij} = \frac{1}{n}\mathop \sum \limits_{t = 1}^{n} \left( {mortality, \;m^{3} } \right)_{tij}$$

where *t *= *year, n *= *number of years simulated, i *= *climate change scenario, j *= *landscape structure scenario.*

The mortality rate includes natural disturbances by wind and bark beetles as well as individual tree mortality from competition or climatic stress (e.g., drought). Also regular timber harvest was included in our impact indicator to account for the total annual removal of Norway spruce in each scenario. The agent-based harvesting module in iLand (Rammer and Seidl 2015) takes into account the natural disturbances in the harvest planning within the preset harvest quotas and thus the timber harvest is at least partly regulated by natural disturbance pulses.

The recovery capacity of forests is frequently assessed as their ability to regenerate after mortality events (Harvey et al. 2016; Hansen et al. 2018). As managers strongly control regeneration mechanisms via planting in our study area, and as we use this control mechanism to design a wide variety of orthogonal scenarios of landscape composition and configuration (Fig. 1b), focusing on tree regeneration as a response variable was not meaningful in our analysis. Once trees have regenerated, whether they are able to grow is an important determinant of ecosystem recovery. Consequently, we focused on mean annual growth of Norway spruce (m^3^ year^−1^) in the different composition and configuration scenarios to assess their recovery ability under climate change (Eq. 4).

4 $$Recovery_{ij} = \frac{1}{n}\mathop \sum \limits_{t = 1}^{n} \left( {growth,\; m^{3} } \right)_{tij}$$

Ingrisch and Bahn (2018) argue for the normalization of both impact and recovery measures relative to a meaningful baseline when assessing resilience. We used the simulated Norway spruce growing stock under a continuation of historic climate conditions (Table S3) as baseline for calculating climate change effects, deriving separate baselines for each composition and configuration scenario (Eqs. 5–7).

5 $$Baseline_{j} = \frac{1}{n}\mathop \sum \limits_{t = 1}^{n} \left( {growing\;stock\;under\;historic\;climate, m^{3} } \right)_{tj}$$

6 $$RelRecovery_{ij} = \frac{{Recovery_{ij} }}{{Baseline_{j} }}$$

7 $$RelImpact_{ij} = \frac{{Impact_{ij} }}{{Baseline_{j} }}$$

Norway spruce resilience to climate change was calculated as a net effect between *RelRecovery* and *RelImpact* (Eq. 8). In other words, we define resilience as the delta between growth and mortality, relative to baseline conditions.

8 $$Resilience_{ij} = RelRecovery_{ij} - RelImpact_{ij}$$

Our resilience indicator thus expresses the viability of the species under climate change by assessing whether it is able to maintain its growing stock in the face of climate-mediated changes in tree mortality and growth. We deemed Norway spruce to be resilient when the relative recovery capacity compensated or overcompensated the relative impacts, i.e. when Eq. (8) resulted in positive values. All analyses were done using R (R Development Core Team 2017).

## Results

### Natural disturbance processes

**Fig. 2:**
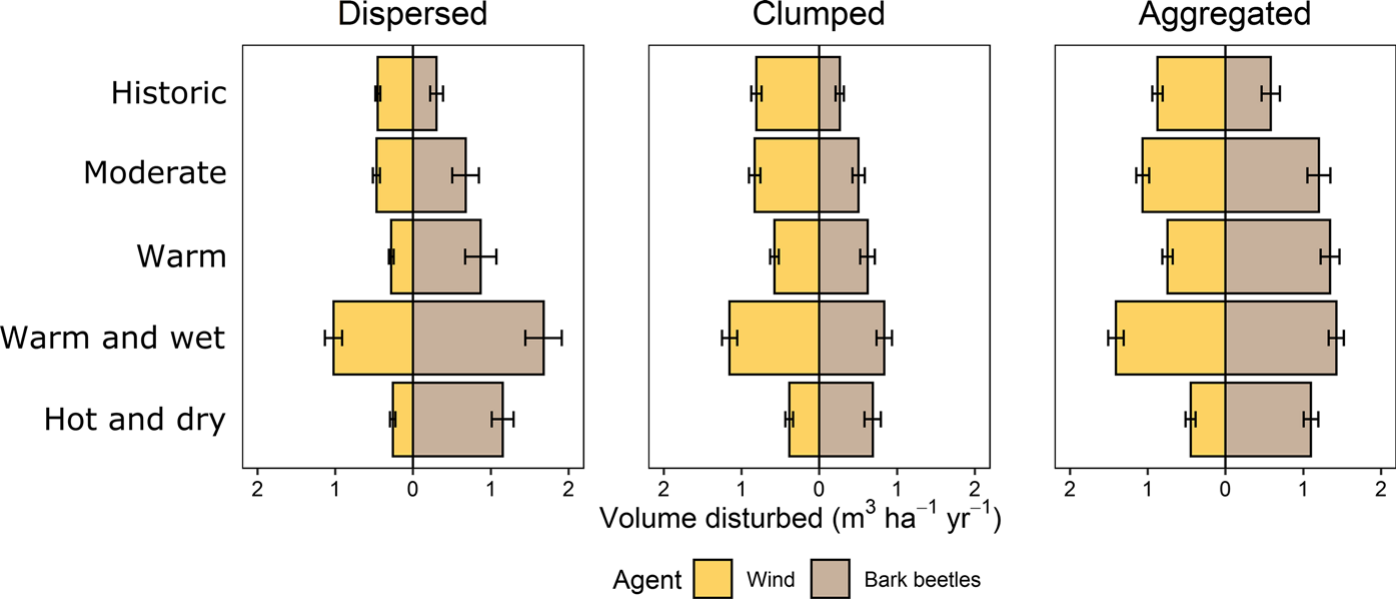
Mean annual Norway spruce timber volume disturbed by wind and bark beetles in different spatial configurations (panels) and climate scenarios (rows). Values are reported for a Norway spruce percentage of 30% on the landscape, and are averages over the 190 year simulation period. Whiskers indicate the range between replicated simulations

Landscape configuration had a strong effect on wind and bark beetle disturbances (Fig. 2). The scenario with *aggregated* Norway spruce stands was most vulnerable to wind and bark beetle disturbances, regardless of climate and composition scenario (Fig. 2, Table S4). Dispersing Norway spruce throughout the landscape resulted in the lowest level of wind disturbance, but bark beetle spread was contained well also in the *clumped* scenario (Fig. 2, Table S4). Increased stand stability and reduced edge density in the mixed stands reduced wind risk in the *dispersed* scenario whereas increased distances between potential host trees (dilution effect) reduced bark beetle outbreaks. Two further insights emerged from comparing the two scenarios featuring monospecific spruce stands, namely the *clumped* and *aggregated* scenarios (Table 2). First, the mean and maximum area disturbed by wind did not differ much between these scenarios, suggesting that monospecific Norway spruce stands were vulnerable to wind damage regardless of their location in the landscape. Second, the mean annual area infested by bark beetles was lower in the *clumped* configuration compared to the *aggregated* scenario. Also, when Norway spruce percentage was < 30%, the mean annual infestation area was smaller than the average Norway spruce patch size in the *clumped* scenario.

**Table 2:** Mean area disturbed per disturbance agent

Disturbance agent	Composition (%)	Mean patch size of aggregated Norway spruce stands (ha)			Configuration					
					*Dispersed*		*Clumped*		*Aggregated*	
		*Dispersed*	*Clumped*	*Aggregated*	Mean	Max	Mean	Max	Mean	Max
Wind (disturbed area ha year^−1^)	10	–	3.2	670.3	1.9	99.7	2.4	134.9	2.5	127.2
	20	–	5.2	1340.8	2.5	112.2	3.4	201.1	4.2	214
	30	–	8.2	2011.2	3	140.2	4.8	294.7	5.8	292.3
	40	–	11.2	2681.5	3.6	180	6.2	393.2	7.3	355.9
	50	–	13.2	3351.9	4.6	255.2	7	445.9	8.9	437.8
Bark beetles (infested area ha year^−1^)	10	–	3.2	670.3	4.7	109.7	1.7	19.2	5.2	55.7
	20	–	5.2	1340.8	9.2	149	3.9	52.8	13.1	141.9
	30	–	8.2	2011.2	13.4	226.2	7.8	118.1	19.4	199
	40	–	11.2	2681.5	17.8	340.1	12.5	180.4	25.1	265.7
	50	–	13.2	3351.9	22.2	387.2	15.7	255.3	29.9	338.4

Data are aggregated over all climate scenarios. Mean patch size of aggregated Norway spruce stands for comparison (i.e. mean area of clumps for *clumped* scenario and the total spruce area for the *aggregated* scenario)

Climate change had a strong impact on wind disturbance. Both the warm and the hot and dry scenario had considerably fewer storm events (Table 1), resulting in 38% and 43% lower timber volume disturbed compared to wind disturbances under historic climate (Fig. 2). Climate change also altered the relative importance of wind and bark beetle disturbances: While wind disturbances were higher than the bark beetle disturbances under historic climate, climate change had a strong positive effect on bark beetles, which reached levels equal to or exceeding wind disturbances under many climate change scenarios.

**Fig. 3:**
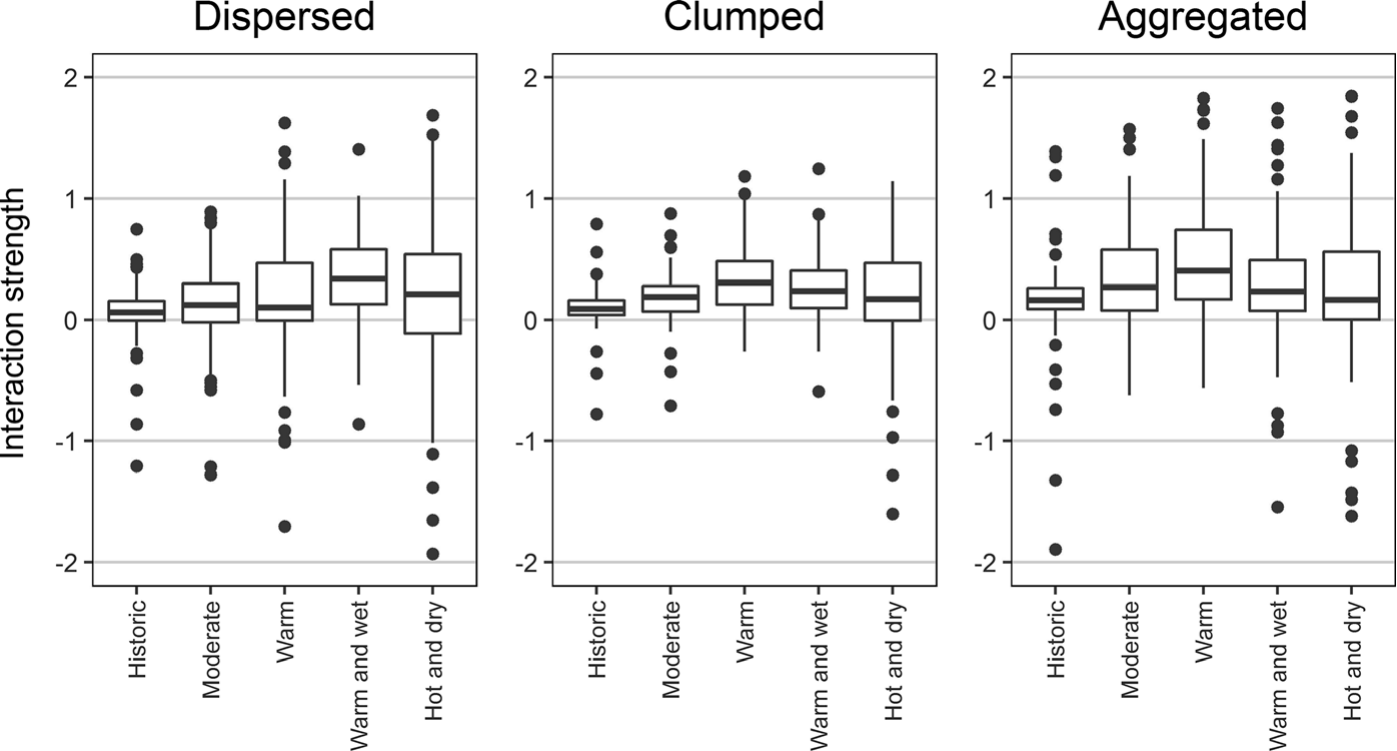
The interaction strength between wind and bark beetle disturbances in different spatial configurations (panels) and climate scenarios (boxes). Values are reported for a Norway spruce percentage of 30% on the landscape. Values > 0 indicate positive, amplifying interactions. Y-axes were truncated to improve clarity. Boxes indicate the interquartile range, whiskers extend to the 1st and 99th percentiles, while dots represent outliers beyond these

The interaction effect between wind and bark beetles was positive in all scenarios (i.e., with wind amplifying bark beetle disturbances) and increased with climate change (Fig. 3). The interaction strength was also modulated by landscape configuration. The interaction was strongest in the *aggregated* scenario and weakest in either the *dispersed* or *clumped* scenarios (depending on the climate change scenario studied, Fig. 3). However, under historic climate and moderate climate change there was no clear difference in disturbance interactions between spatial configurations.

Increasing percentages of Norway spruce amplified both wind and bark beetle disturbances, but disproportionally increased bark beetle disturbances (Table S4). For example, an increase of Norway spruce percentage from 10 to 50% on the landscape under moderate climate change increased the wind disturbance volume by + 184.9%, + 229.7% and + 271.9% in *dispersed*, *clumped* and *aggregated* configurations, respectively. The corresponding increase in bark beetle disturbances was + 1002.8%, + 1048.9% and + 401.5%. Bark beetle susceptibility thus responded more strongly to increasing host tree percentages in *dispersed* and *clumped* scenarios, while the absolute levels of bark beetle disturbance were highest in the *aggregated* configurations (Table S4).

### Resilience to climate change

Both landscape configuration and composition influenced Norway spruce resilience to climate change. Landscape configuration had a strong effect on the impact and recovery of Norway spruce in the landscape over the simulation period (Fig. S8). Dispersing Norway spruce in mixed stands favored the recovery of growing stock. However, higher growing stock levels also—with a time lag of several decades—led to increased climate change impacts. We thus found considerable feedbacks between the two resilience dimensions, with higher recovery level priming the system to subsequent higher impacts. In the *clumped* and *aggregated* scenarios climate change impacts were strongly dominated by mortality pulses from individual disturbance events. Also, very high mortality in the first decades of the simulation led to decreasing growth in the warm and dry scenario under *clumped* and *aggregated* configurations. The *dispersed* scenario was more buffered with regard to both of these aspects (Fig. S8).

**Fig. 4:**
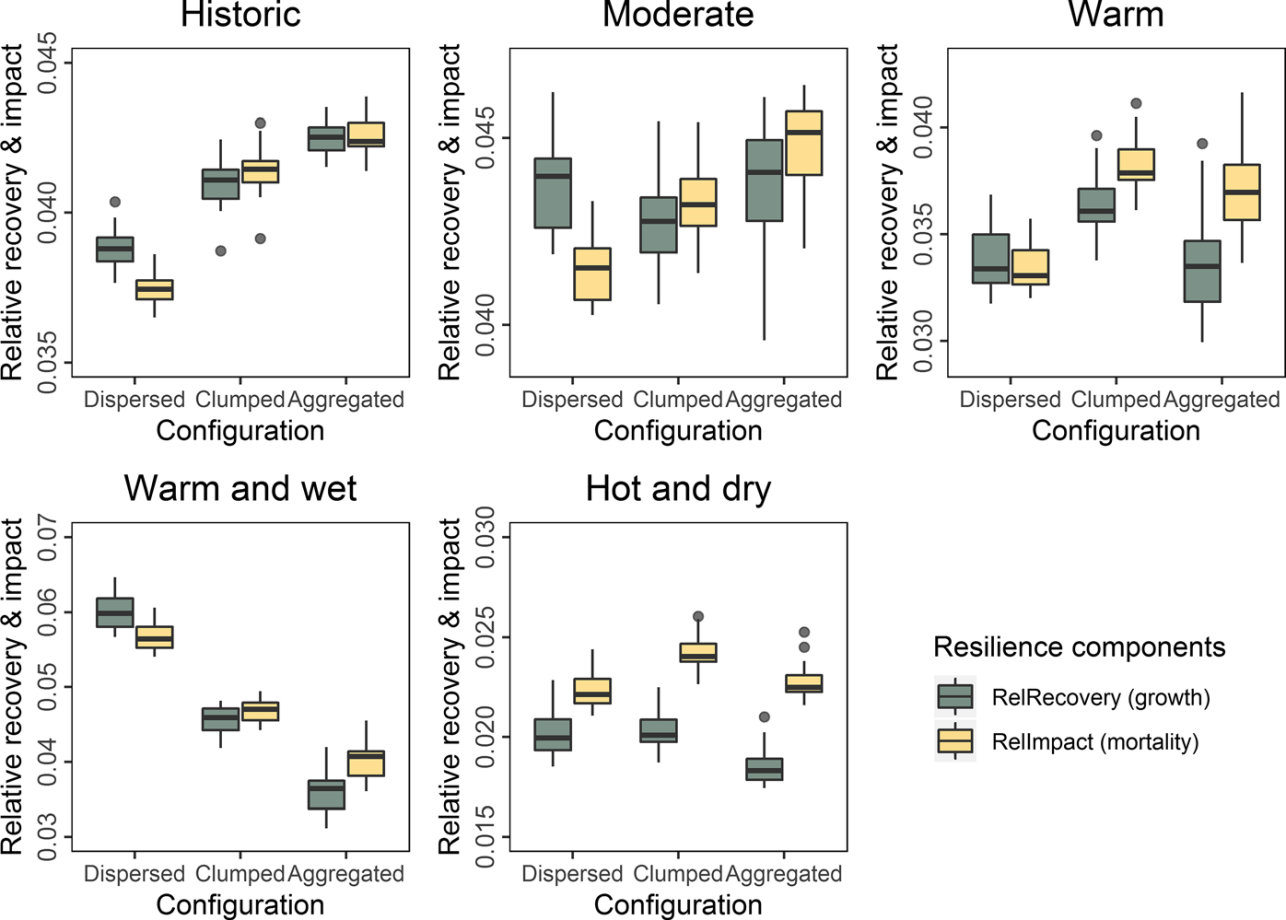
Relative recovery and impact values for a Norway spruce percentage of 30% on the landscape (Green > Brown = Resilience). Each panel depicts the difference between spatial configuration scenarios under one climate scenario. Boxes indicate the interquartile range between the 20 replicates for each scenario, whiskers extend to the 1% and 99% quantiles. Note that y-axes are scaled differently

Interestingly the *aggregated* configuration had the highest relative recovery and impact values under historic climate. Increasing natural disturbances under climate change reversed this ranking, with the *dispersed* and *clumped* scenarios having higher values especially under the RCP 8.5 scenarios (Fig. 4). Also, the balance between recovery and impacts (here defined as the resilience of the system, Eq. 8) was increasingly shifted towards impacts outweighing recovery under climate change in the *aggregated* configuration. In contrast, the *dispersed* scenario had the strongest positive difference between recovery and impact, making it the overall most resilience landscape configuration. These analyses underline that our findings regarding resilience are more strongly driven by the differential responses of climate change impacts and recovery than by the absolute changes in either one of these indicators.

**Fig. 5:**
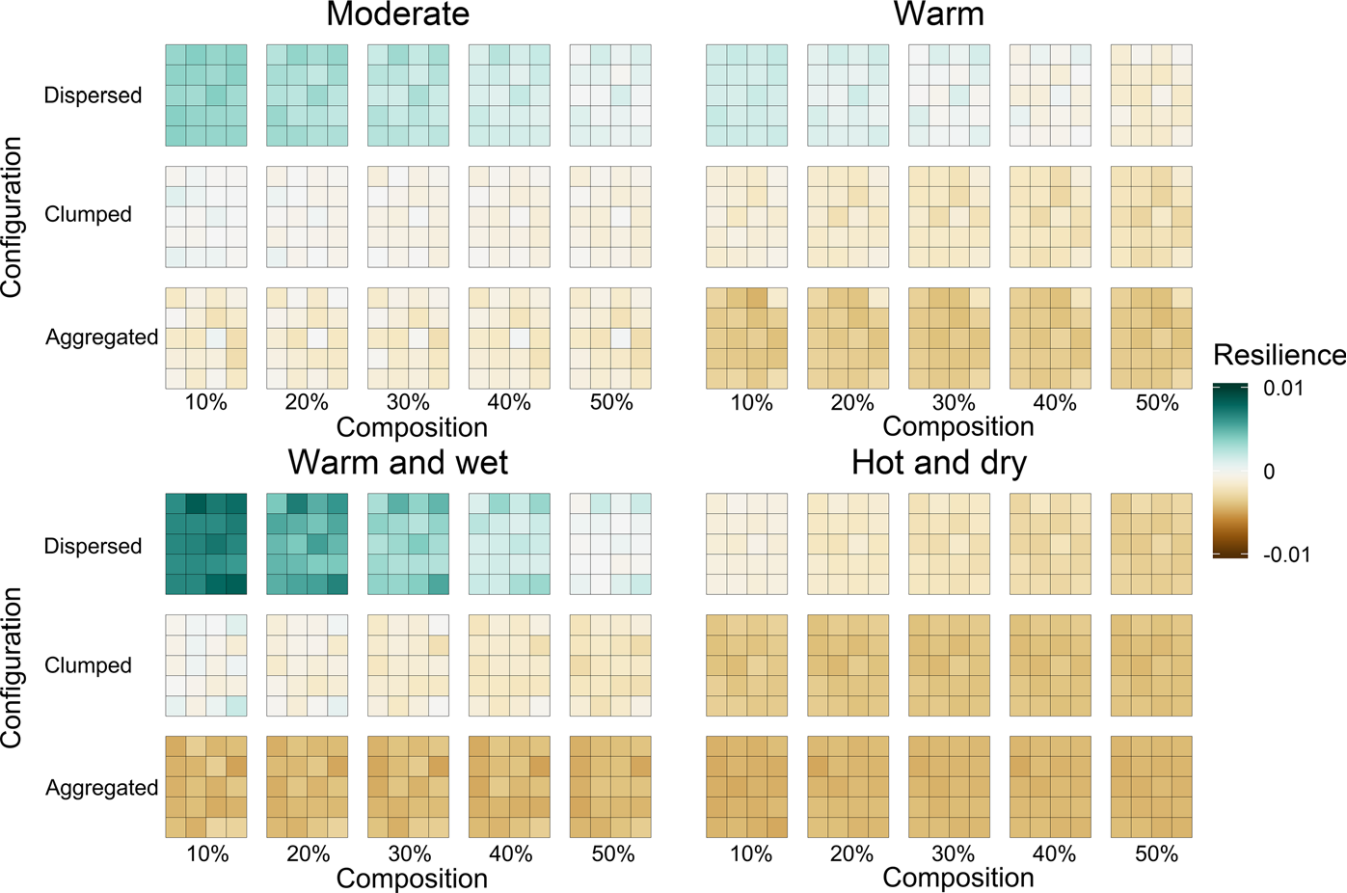
The effects of landscape configuration and composition on Norway spruce resilience to climate change. Resilience is expressed as the net change between relative recovery and impact over the 190 year simulation period. Positive values (green tones) indicate higher resilience and negative values (brown tones) lower resilience. Each pane represents a different climate change scenario and each large tile a single configuration—species scenario, consisting of the values for 20 replicated simulations (indicated by individual pixels) and thus illustrating the uncertainty in the simulations

The effects of spatial configuration increased with decreasing Norway spruce percentage. Recovery and impact relative to baseline values did not vary strongly with composition within the same climate change scenario (Table S2). In contrast, Norway spruce resilience to climate change (i.e. the net effect between relative recovery and impact) decreased with increasing spatial aggregation as well as with increasing Norway spruce percentage (Fig. 5). The effects of spatial configuration were stronger than those of species composition. In *dispersed* configurations, Norway spruce was resilient to climate change scenarios except under the most extreme hot and dry scenario. Conversely, when *aggregated* on the landscape, Norway spruce was never resilient to climate change (Fig. 5). The *clumped* scenario was in between the *dispersed* and *aggregated* scenarios, with negative resilience in the warm as well as hot and dry climate scenarios, and no clear signal in the moderate and warm and wet scenarios. Our numeric values of resilience were relatively small [− 0.01 to 0.01], which is the effect of (i) benchmarking all values of change against average growing stock values (Eqs. 5–7), with fluxes being usually orders of magnitude lower than stocks in forest ecosystems (Waring and Running 2007), and (ii) defining resilience as the delta between two interacting variables. We note that the underlying trajectories of tree growth and mortality change by as much as + 180% and − 99% (Fig. S8), and that even small differences in the balance between climate change impacts and the ability to recover from them can result in ecologically significant changes.

## Discussion

We presented a quantitative assessment of the effects of landscape configuration and composition on forest resilience to the combined influences of biotic and abiotic disturbances under various future climate scenarios. Our simulation results suggest that both landscape configuration and composition influence the resilience of Norway spruce to climate change at the trailing edge of its distribution. We further showed that the effects of landscape configuration are stronger than those of landscape composition. In line with our initial hypothesis, Norway spruce was most resilient to climate change when planted in mixed-species stands. Our results are consistent with the growing evidence from empirical and experimental studies on the positive effects of mixed forests under climate change (Bauhus et al. 2017; Jactel et al. 2018). Furthermore, our study is in agreement with previous assessments showing high climate sensitivity of Norway spruce, particularly at the trailing edge of its distribution (Boden et al. 2014; Seidl et al. 2017b).

Our findings of strong landscape-scale drivers of resilience are important as they complement previous assessments of Norway spruce resilience at the tree and stand scale, identifying stand age and density (Seidl et al. 2017b) as well as site conditions, most notably water availability (Boden et al. 2014; Zang et al. 2014) as the key drivers of resilience to climate change. Consequently, the consideration of multiple spatial scales is crucial for a comprehensive assessment of ecosystem resilience (Craven et al. 2016). In this context it is noteworthy that effects of individual processes on resilience can vary on different spatial scales. For example, fire can reduce the resilience of individual stands, but increase the landscape resilience due to changes in the landscape configuration and composition (Johnstone et al. 2010; Seidl et al. 2016a).

Changing natural disturbance regimes are a major factor challenging the resilience of Norway spruce forests (Seidl et al. 2009). Wind and bark beetle disturbances are expected to increase in the future due to structural changes in forests as well as due to a warming climate (Hanewinkel et al. 2013; Seidl et al. 2014c). Bark beetle disturbances are especially sensitive to climate change as trees could be increasingly stressed during extended drought periods, reducing their capacity to defend against bark beetle attacks (Netherer et al. 2015; Seidl et al. 2016b). Furthermore, warmer temperatures are expected to positively affect the voltinism and population growth rates of important bark beetle species (Jönsson et al. 2011; Økland et al. 2019). Our simulation results confirm these expectations, with increasing disturbances in all future scenarios compared to simulations under historic climate, and a particularly strong response of bark beetle disturbances to climate change (Fig. 2). The climate scenario most strongly affected by disturbances was the warm and wet scenario. This scenario resulted in an initial increase in growing stock in all configurations. In addition, the high precipitation together with a large number of wind events (Table 1) resulted in massive wind disturbance events, with soil wetness decreasing tree anchorage and tall trees being more vulnerable to wind disturbance (Peltola et al. 1999; Mitchell 2013; Seidl et al. 2014a). Furthermore, our results highlight that climate change could increase the interaction strength between wind and bark beetle disturbances (Fig. 3). Disturbance interactions thus contribute considerably to the climate sensitivity of natural disturbances (Seidl and Rammer 2017; Lucash et al. 2018). We for the first time showed that the widely reported interaction between wind and bark beetle disturbances (Eriksson et al. 2005; Stadelmann et al. 2014) is modulated also by landscape configuration. In the particular context of bark beetle disturbances, the connectivity between host and bark beetle populations has previously been highlighted as a key driver for large scale outbreaks (Raffa et al. 2008; Seidl et al. 2016b). In line with our hypothesis we found *clumped* configurations of host trees to have a dilution effect on bark beetles, reducing the timber volume disturbed by bark beetles. Bark beetle disturbances in *clumped* monospecific Norway spruce stands were even lower than in mixed stands, underlining that for insects with short dispersal range, such as bark beetles, local host availability and connectivity are more important factors than tree species diversity in general (Jactel et al. 2017). A further refinement of reducing beetle risk through *clumped* configurations would be to also consider the specific age of the *clumped* Norway spruce cohort in the design of the configuration. As bark beetle susceptibility is low in young stands a high age variation in neighboring clumps of potential host tree species could further contain bark beetle outbreaks and increase landscape resilience.

We note that we did not assess the ecological resilience of the entire forest ecosystem in the Bucklige Welt study area. We rather focused on the resilience of a single species, Norway spruce, to changing climatic conditions. This type of analysis is complementary to more comprehensive assessments of ecological resilience, and allows a better understanding of the specific processes affecting resilience to emerge (Buma and Wessman 2012; Hansen et al. 2018). With regard to determining underlying processes our analysis highlights that the recently proposed resilience framework by Ingrisch and Bahn (2018) has limitations if impact and recovery processes are not independent of each other (as is the case with tree growth and mortality). We were thus not conclusively able to assess whether trends in Norway spruce resilience stem primarily from changes in impact (i.e. mortality) or recovery (i.e. growth). However, in depth analysis of temporal trajectories indicated that the results were driven by an initially positive growth signal that was overcompensated by increasing mortality in the later decades of the simulation. Notwithstanding the socioeconomic importance of Norway spruce—being the main source of income for local forest owners and forming the backbone of the local wood processing industry—future analyses should extend the scope to a full ecosystems perspective. Another limitation of our study is that it is solely based on results of simulation modeling. To increase the confidence in our simulation results we conducted a pattern-oriented model evaluation against independent data (e.g. disturbance data and growth and yield information from the BOKU school forest), finding good correspondence between simulated and observed patterns in our study landscape. Nonetheless, augmenting simulation studies with experimental approaches would be desirable. In this regard it is important to note, however, that manipulating factors such as landscape configuration is virtually impossible in experiments (e.g., due to the high costs involved as well as the inability to replicate landscapes, Phillips 2007). Simulation models like the one applied here are thus important tools for making inferences at scales beyond the stand scale (Shifley et al. 2017).

Several important implications for ecosystem management arise from our results: We clearly showed that managing for pure Norway spruce stands at low elevations is not resilient under climate change. This result was particularly driven by strongly increasing natural disturbances from wind and bark beetles. Consequently, adaptation measures are needed in Norway spruce forests at the trailing edge (Lindner et al. 2010; Hlásny et al. 2017). The past research in this regard has largely focused on stand level measures such as thinning (Elkin et al. 2015), or changing the species composition away from Norway spruce altogether (Jactel et al. 2009). Our study confirms that increasing tree species diversity at stand scale increases the resilience of Norway spruce to climate change. However, we demonstrate that landscape-scale approaches such as modifying the landscape configuration and composition are potent approaches for increasing Norway spruce resilience. Our simulation results suggest that while reducing the percentage of Norway spruce on the landscape generally increased the resilience of the remaining Norway spruces, also *clumped* or *dispersed* configurations increase resilience over the management of large blocks of the species on the landscape. Should management desire to maintain substantial Norway spruce percentages, disaggregation approaches at the landscape scale are thus recommended. We note that the implication of a concerted landscape-scale management is complicated by a small-scale owner structure in many parts of Central Europe. However, owners associations could facilitate joint management plans of neighboring small-scale owners in order to shape landscape structure in a coordinated manner. In this regard it is important to stress that such advances could not only foster the resilience of economically important species such as Norway spruce, but could also have benefits for biodiversity, such as favoring beta diversity and preserving the connectivity of key habitats for multiple species groups (Lindenmayer and Franklin 2002; Mori et al. 2018; Schall et al. 2018; Seibold et al. 2019). We conclude that landscape composition and configuration are important drivers of forest resilience. Given that forest resilience is increasingly challenged by climate change, landscape composition and configuration should thus receive increased attention in ecosystem management.

## Electronic supplementary material

Below is the link to the electronic supplementary material.

Supplementary material (DOCX 2798 kb)
