# Supplementary material for: Norway spruce at the trailing edge: the effect of landscape configuration and composition on climate resilience
Source: Landsc Ecol. 2020 Jan 11;35(3):591–606. doi: 10.1007/s10980-019-00964-y (PMC7081663; doi:10.1007/s10980-019-00964-y)
Supplement: Supplementary file 1 — Supplementary material (DOCX 2798 kb) [file 10980_2019_964_MOESM1_ESM.docx]

*Supplementary material*

**Norway spruce at the trailing edge:**

**The effect of landscape configuration and composition on climate resilience**

Juha Honkaniemi*, Werner Rammer, Rupert Seidl

Institute of Silviculture, University of Natural Resources and Life Sciences (BOKU), Peter-Jordan Strasse 82, 1190 Vienna, Austria

* correspondence to: [juha.honkaniemi@boku.ac.at](mailto:juha.honkaniemi@boku.ac.at) ([+43 1 47654-91355](tel:+43%201%2047654-91355))

**Supplementary Material S1: Model evaluation**

***Productivity analysis***

The simulated productivity of different species in the landscape was evaluated against data from BOKU school forest. The 1135 ha of forest in the school forest is divided into stands ranging in area from 0.1 – 11.5 ha with a mean size of a stand at 1.2 ha including detailed vegetation information. A total of 1230 resource units (100m resolution) of our study landscape with climate and soil data were fully or partially intersecting with the school forest stand polygons. Stands were in many cases mixed species stands with several different tree species in various layers. For the productivity simulations, we chose from each stand all the tree species in the dominant canopy layer. Stand data per tree species was assigned to resource units. Stand data that covered the largest area of the resource unit, was assigned for that resource unit. In case the stand had several tree species in the dominant canopy layer, the resource unit environmental data (climate and soil) was copied for each tree species. Tree species with less than 15 simulated resource units were omitted before the simulations. In addition, Douglas fir (n=41) was omitted as there was no proper yield table available for the analysis. This yielded in total of 4019 resource units to be simulated as monospecific stands according to the yield tables.

Site indices in Austria are based on yield tables, where thinnings are carried out every 10 years reducing the stem number in the stand. The site indices are then based on the dimensions of 100 tallest trees at 100 years of stand age. For each resource unit we had the following data for simulations:

- Climate and soil data
- Tree species (each resource unit simulated as a monospecific stand)
- Site index from BOKU school forest inventory data

At the initialization of the test, the stands were set to be 40 years old and the initial tree conditions were derived from yield tables based on the site indices from BOKU school forest. Correct yield table was chosen and the stem number at 40 years chosen for each unit depending on the tree species and the site index. In iLand, regeneration and mortality modules were disabled and the stem number of the stands was controlled by removing trees every ten years in the middle of a decade (45, 55, 65, …, 95 years) according to the yield table. Climate data for this simulation was randomly sampled from EC-EARTH and KNMI-RACMO22E RCP4.5 data from period of 1981-2010 (i.e. historic climate). Resource units where run independently from each other (however, assuming themselves to continue homogenously outside the simulated 1 ha area to avoid edge effects) for 60 years to reach a stand age of 100 years.

In general, the simulated results slightly overestimated the productivity compared to the values from yield tables. Therefore, we decreased the amount of available nitrogen in all resource units by 5 kg ha^-1^ throughout the whole landscape. With this reduction, the overall fit was good (Fig. S1, Table S1).


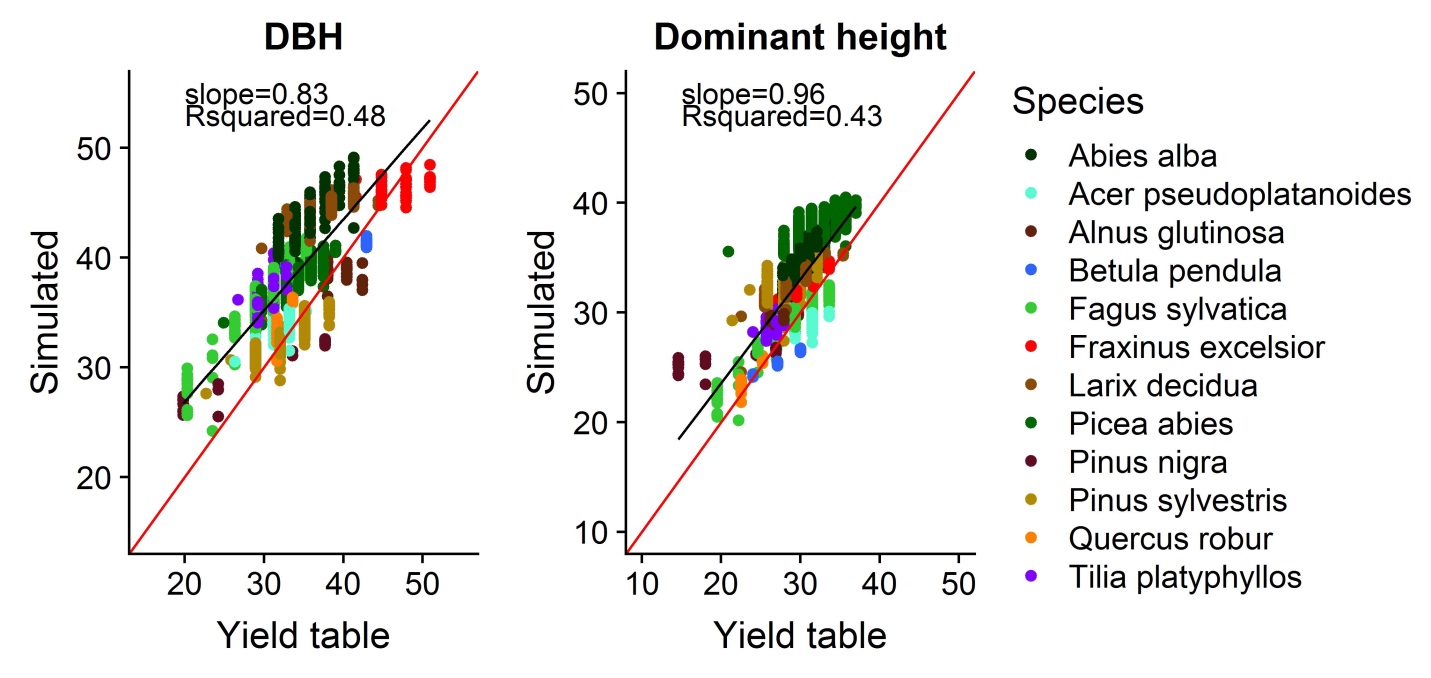


**Fig. S1**. Average diameter at breast height (DBH) and dominant height of the simulated unit compared against the yield table values for all tree species. Linear regression models show the fit of the data against the yield table values.

**Table S1.** Productivity analysis results for DBH and dominant height all the tree species. Regression models were produced for all the species in the productivity analysis based on the data presented in Fig. S1.

|  |  | **DBH** | | **dominant height** | |
| --- | --- | --- | --- | --- | --- |
| **Species** | N | slope | R^2^ | slope | R^2^ |
| *Abies alba* | 224 | 0.70 | 0.73 | 0.97 | 0.80 |
| *Acer pseudoplatanus* | 89 | 0.50 | 0.48 | 0.29 | 0.42 |
| *Alnus glutinosa* | 47 | -0.10 | 0.05 | 0.27 | 0.46 |
| *Betula pendula* | 33 | 0.25 | 0.91 | 0.40 | 0.98 |
| *Fagus sylvatica* | 1285 | 0.85 | 0.85 | 0.64 | 0.89 |
| *Fraxinus excelsior* | 59 | 0.10 | 0.09 | 0.54 | 0.90 |
| *Larix decidua* | 739 | 0.29 | 0.52 | 0.52 | 0.86 |
| *Picea abies* | 1182 | 0.54 | 0.75 | 0.36 | 0.47 |
| *Pinus nigra* | 37 | 0.32 | 0.94 | 0.13 | 0.61 |
| *Pinus sylvestris* | 769 | 0.51 | 0.76 | 0.43 | 0.33 |
| *Quercus robur* | 40 | 1.55 | 0.75 | 0.85 | 0.84 |

One explanation for the overestimation could be that the trees measured for the yield tables grew in different climate conditions compared to our simulated trees with recent climate (random sampling of climate data from time period 1981-2010). Therefore, we compared our results to corresponding field data (Pretzsch et al., 2014). This analysis showed that the simulated productivity was well in line with field data although still slightly overestimating it on the poorer sites for both main species of European beech and Norway spruce (Fig. S2).


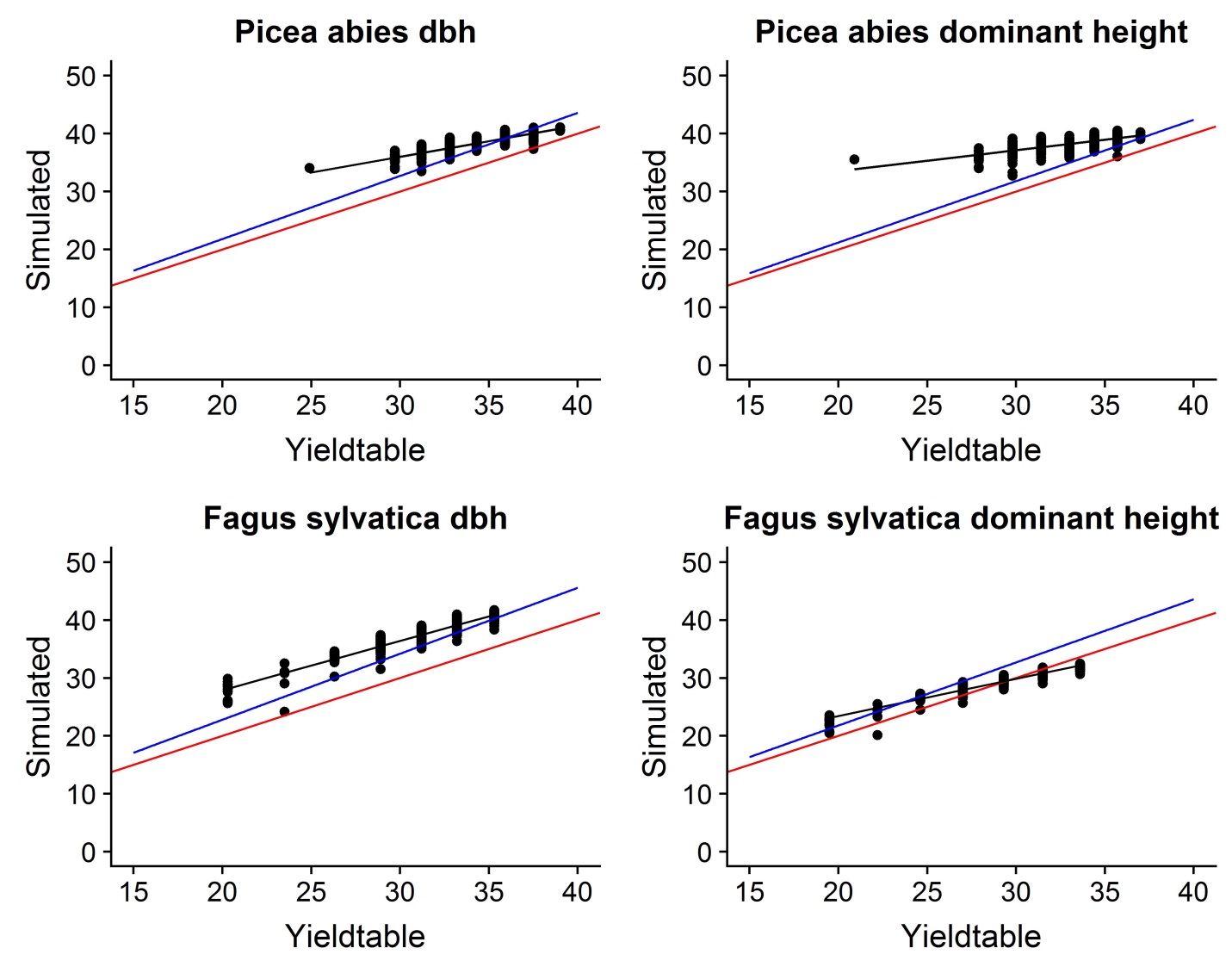


**Fig. S2***.* Average diameter at breast height (DBH) and dominant height of the simulated unit compared against the yield table values for two of the most dominant species of the landscape; European beech and Norway spruce. Dots are the simulated units and the black line is a simple linear regression line. Red line is the 1:1 line between simulated and yield table values. Blue line is derived from (Pretzsch et al., 2014).

***Potential natural vegetation (PNV) composition***

The potential natural vegetation (PNV) of the landscape was analyzed by simulating forest dynamics from bare ground for 1000 years without management and natural disturbances. Seeds of all tree species were assumed to be dispersed in the landscape evenly for natural regeneration. Climate data was randomly sampled from a period of 1981-2010 of the EC-EARTH and KNMI-RACMO22E RCP4.5 scenario for historic climate as described above.

The results show that the PNV of the landscape is dominated by European beech (*Fagus sylvatica*) and silver fir (*Abies alba*) with small share of Norway spruce (*Picea abies*) (Fig. S3). In addition, we classified spatially the PNV results on a resource unit level (100m resolution) mostly as a Beech forest according to the Starlinger-PNV types (see Lexer, 2001) (Fig. S4A). The comparison against the PNV classification for BOKU school forest (Fig. S4B) shows that iLand did not catch all the details and that the Silver fir and Norway spruce shares were too low to classify the forests as Spruce-Fir-Beech forests. However, the distribution maps for the three most dominant species European beech, Silver fir and Norway spruce show that the distribution of the conifers was as expected them being more prevalent at the higher altitudes (Fig. S5). The most likely reason for this is that the climate series that we used as historic climate is already warmer than what the real historic climate would be (N.B. see above the productivity analysis evaluation).


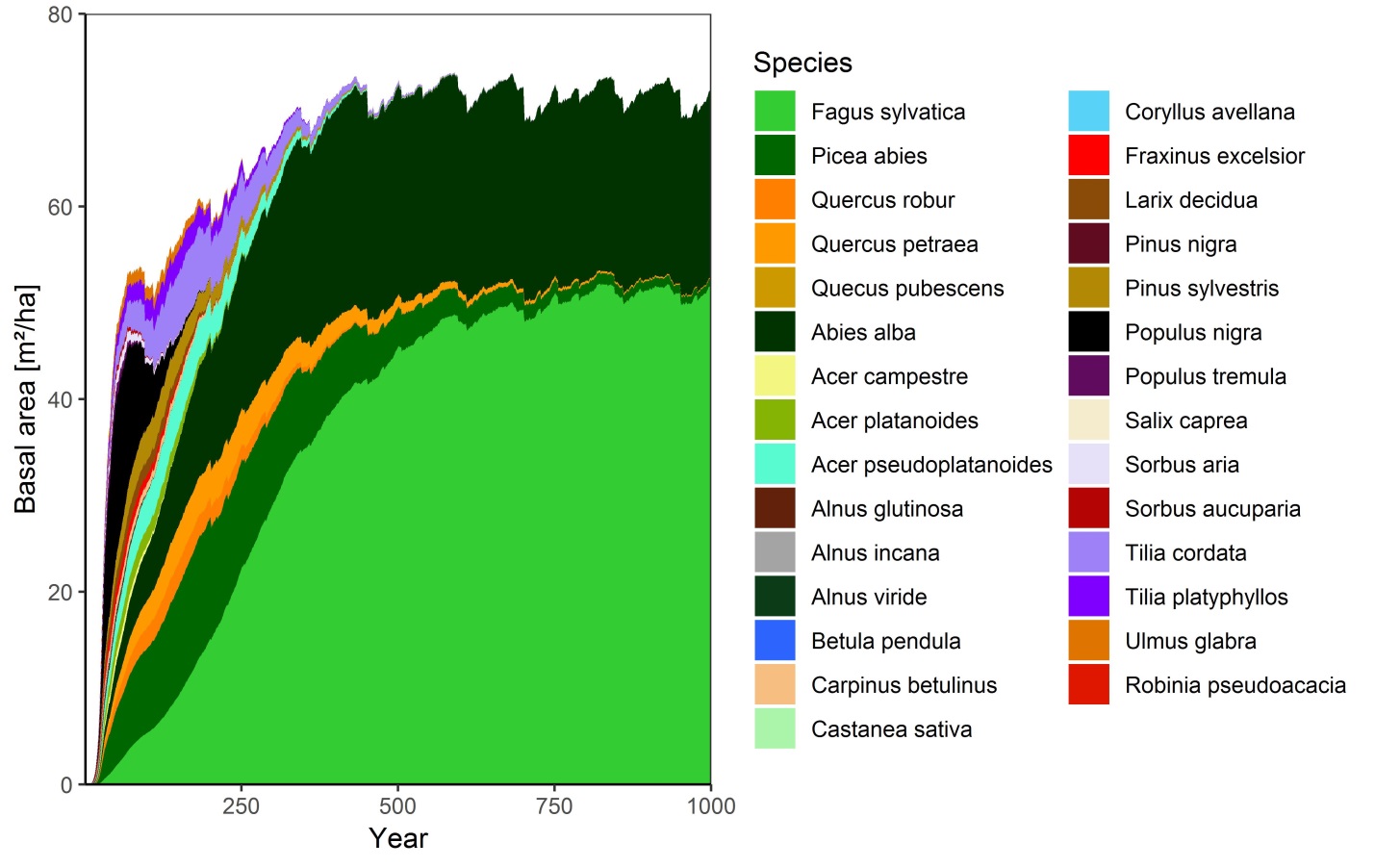


**Fig. S3.** Species share of the total mean basal area in the landscape.


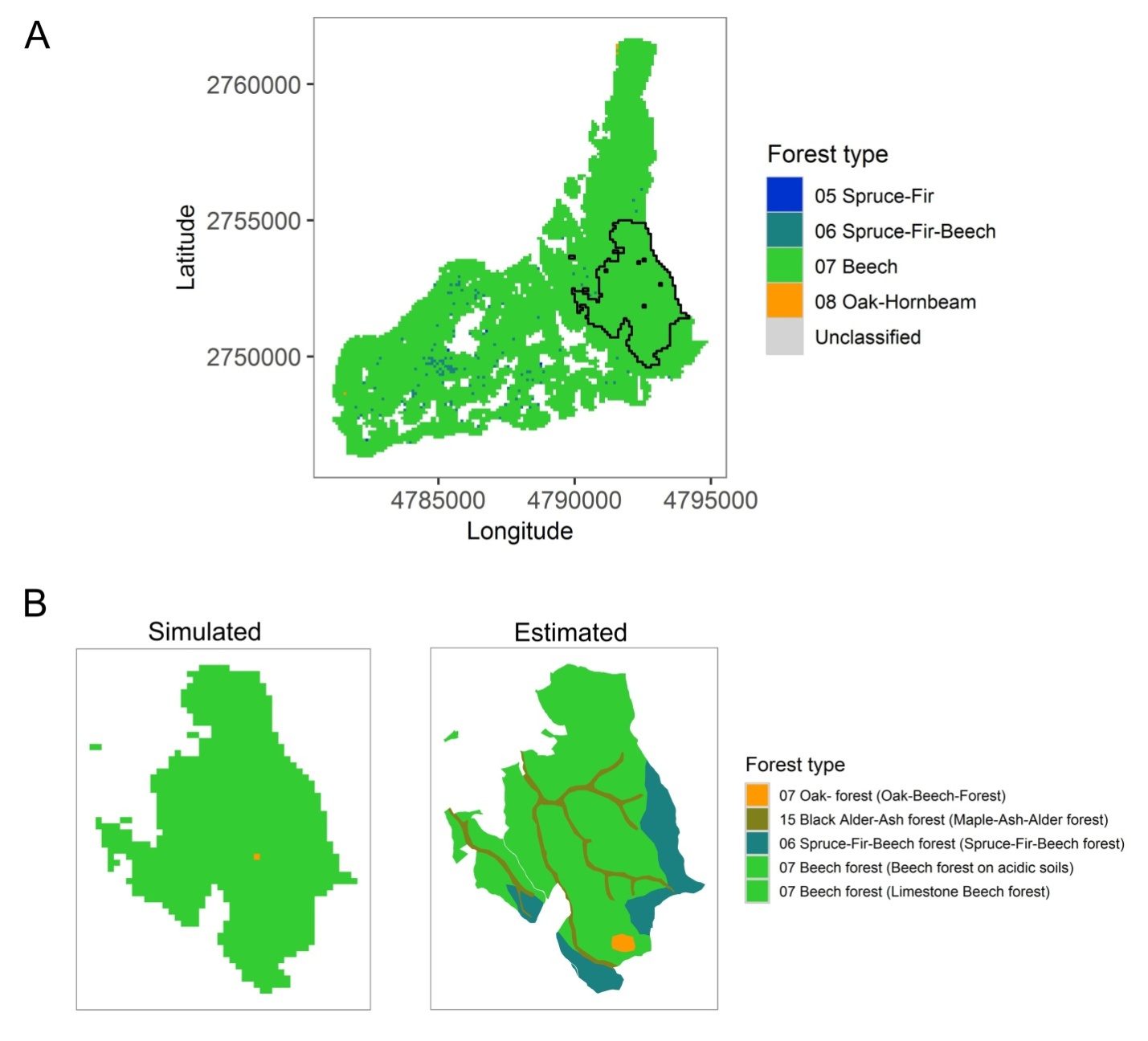


**Fig. S4**. Spatial classification of the PNV results for (A) the whole study landscape and (B) the BOKU school forest. The BOKU school forest classification was evaluated against an estimated, more detailed PNV map for the area.


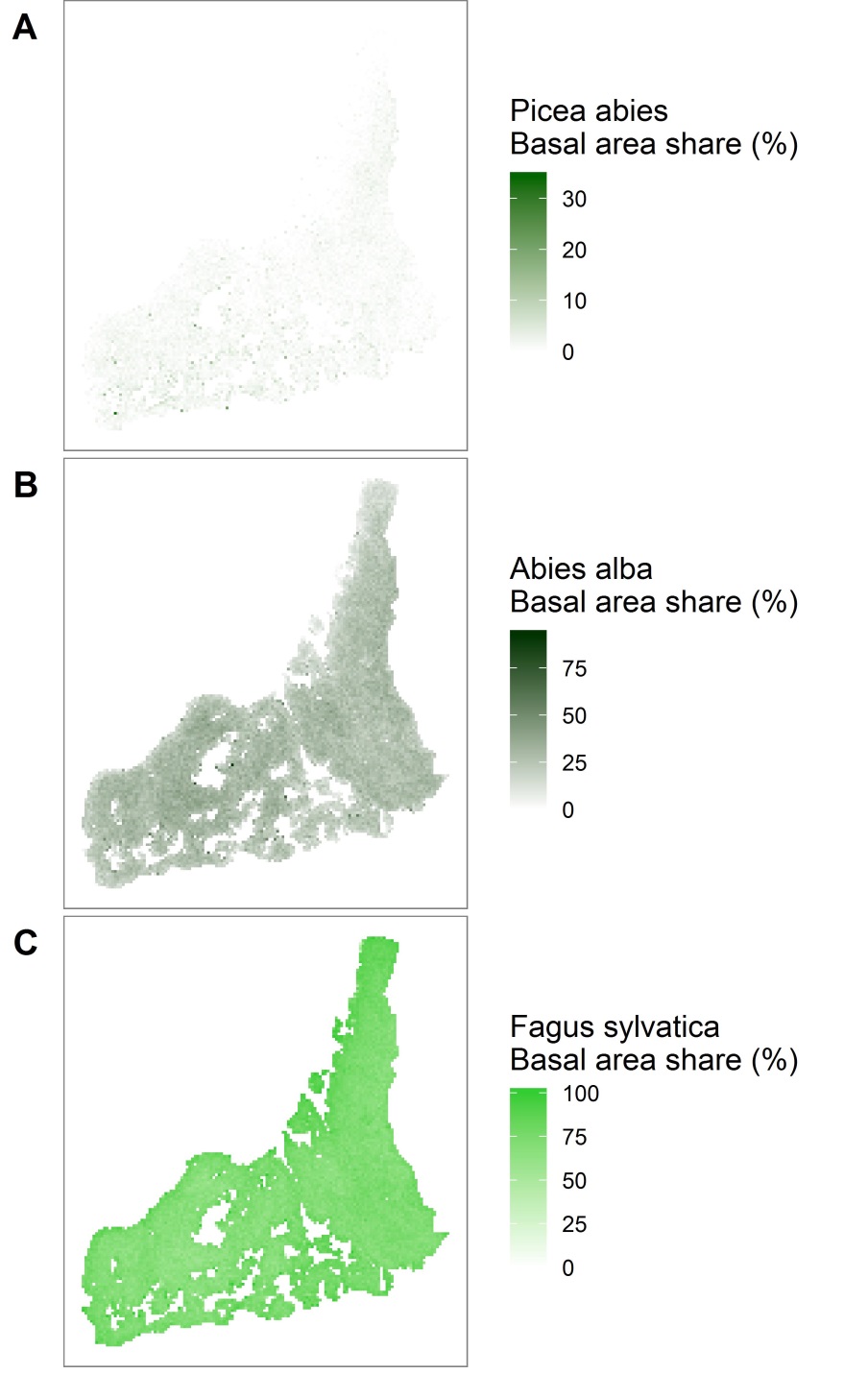


**Fig. S5** Distribution of the three most dominant tree species in the landscape

***Natural disturbances***

Disturbance data was derived from BOKU school forest salvage loggings for a reference period of 2003–2015. The salvage data for BOKU school forest included for that time period one wind storm event, Paula on January 27th 2008. We simulated this event with iLand with a wind speed of 13.40 m/s for the event which was derived from wind gust data from the INCA (Integrated Nowcasting Comprehensive Analysis) dataset (Haiden et al., 2011) (97th percentile of the maximum gust speed for the day of the event of the 1km cells covering the Bucklige Welt). The INCA dataset from the Austrian official weather service (ZAMG) combines several types of model and observational data with detailed digital terrain models on high spatial (1km) and temporal (hourly) resolution for Austria. Duration of the event was estimated to be 3h from 315 degree direction (NW). The simulation resulted in total of 9000 m3 of wind damaged timber with an assumption of 95% efficacy for salvage logging. The comparable wind damaged timber value for BOKU school forest was 10300 m3. The weakness of the wind module calibration in this study is that we had data for only one storm event and only from a part of the simulated area, which though represents well the area. Therefore, the effect of topography to wind speed might not be calibrated precisely. Including data (if available) from other storm events with different wind speed and direction together with wind disturbance data from other parts of the landscape would make the calibration stronger.

Bark beetle module in iLand (<http://iland.boku.ac.at/barkbeetle+module>) requires an initial value for the the annual probability for the occurrence of a bark beetle outbreak per hectare. During the simulations, this probability will then vary based on climatic variation within the landscape and wind disturbance (increased probability after wind disturbance). We tested the values for this probability based on trial and error comparing simulation results against BOKU school forest data of bark-beetle-disturbed timber volume for the INCA period. Initial value of 0.002 gave the bark beetle damage over the whole period 18000 m3 and the corresponding value for BOKU school forest was 18012 m3. This value was at the peak of an outbreak and the minimum values were 0.0005. Thus, in the simulations for this study we varied the initial value for each replication of a given scenario and randomly draw this from values 0.0005–0.0025.

**Supplementary Material S2: Stand structure and initial vegetation for the simulations**

Detailed stand delineation and composition data was available only for the BOKU school forest, although a national level raster classifying forest types into 6 classes (deciduous, deciduous-mix, conifer-mix, conifer, clearcut, and non-forest) based on Landsat satellite images (30m resolution) was available. In addition, LiDAR data for tree height (5m resolution) was available for the state of Lower Austria. This LiDAR data was used to delineate the landscape into a somewhat homogenous forest operational stand structure. First, the stand size distribution for BOKU school forest was derived to be used as a reference for the delineation for the whole study landscape.

Next, the tree height raster was subjected to a low pass filter and the filtered raster aggregated into 10 m resolution (the minimum resolution of iLand management operations). After this, polygons overlapping with the forest type class raster (forested area) were selected excluding urban areas and fields. This automatic stand delineation produced an overall good match for the BOKU school forest stand size distribution. However, with a too long tail meaning that the automation was not able to detect the small differences in tree height without information on tree species or other more detailed data. Stands larger than 5 hectares still after the automatic delineation were further split into smaller units by following the principles of Voronoi polygons. The number of Voronoi polygon centroids was the rounded polygon area in hectares to be split. Finally, polygons smaller than 0.25 ha were merged to their nearest neighbors based on the stand mean tree height to achieve the final stand structure for the study landscape.

The initial vegetation for the different landscape structure scenarios was derived from the BOKU school forest inventory data. Specifically, we used the locally available tree-level information on stands with main tree species other than Norway spruce (n=125) to generate forest structure and composition without Norway spruce. To generate the above-described combinations of landscape structure for Norway spruce, selected stands were populated with Norway spruce according to the configuration and composition of each scenario. This created the target species composition for each stand in each of the landscape structure scenarios. Each stand was assigned age randomly from an even distribution following the normal forest assumption (stand ages in the landscape vary between 0–100 years).

With each stand having a target species composition and stand age, the spatially explicit tree level structure of the landscape was simulated with iLand using a specific spinup procedure (Thom et al., 2018), where each stand is simulated from bare ground with planting and forest management until the target age is reached and the simulated species composition is saved. The planting procedure for the next rotation is then automatically adjusted to better meet the target composition. If the simulated values of the next iteration are better than the previous ones, the new composition is saved. If not, the original composition remains. Minimum of five iterations was performed for each stand to meet the target values and reach a stable starting point for further simulations.

**Supplementary Material S3: Forest management for simulations**

We applied a common forest management regime across all simulated stands. Stand treatment programs were designed to mimic current forest management of Norway spruce in our study area, featuring 4 thinning interventions and a final cut after a 100 year rotation period (Table S2). After the final cut, stands were regenerated via planting the designated species composition of the respective landscape structure scenario. Also natural regeneration was included in the simulations, but stand initiation was primarily driven by planting. Areas disturbed by wind were salvage harvested, extracting 80 % of the windfelled timber, and trap trees were employed to dampen bark beetle outbreaks. Stand treatment programs were identical between spatial configuration scenarios.

**Table S2.** Forest management regimes for the simulations for both mixed and monospecific stands.

| Management operation | Year | Target | Removal per *dbh* classes  DISPERSED | Removal per *dbh* classes  CLUMPED/AGGREGATED | Constraints |
| --- | --- | --- | --- | --- | --- |
| Young stand tending 1 | 10 | Remove 35% volume | [25,25,25,25] | [25,25,25,25] | *dbh*>5 cm |
| Young stand tending 2 | 20 | Leave 2500 stems | [25,25,25,25] | [25,25,25,25] |  |
| First thinning | 35±5 | Remove 35% volume | [25,25,25,25] | [10,20,40,30,0] | *dbh*>7 cm, *H*>13 m |
| Second thinning | 50±5 | Remove 35% volume | [25,25,25,25] | [10,20,40,30,0] | *dbh*>7 cm, *H*>17 m |
| Third thinning | 60±5 | Remove 15% volume | [25,25,25,25] | [10,20,40,30,0] | *dbh*>7 cm |
| Clearcut | 100±10 |  |  |  | *dbh*>5 cm |

**Supplementary Material S4: Climate data**

Climate data for the study area was derived from different GCM-RCM combinations including daily values between 1981-2100 on a 1km resolution for minimum temperature (*tmin*), maximum temperature (*tmax*), precipitation (*prec*), radiation (*rad*) and vapour-pressure deficit (*vpd*) in 4 different climate change scenarios (see Table 1 in the main paper). In addition to the climate data, each 1km cell included the mean elevation (masl.). No historic climate data was available and thus we used the first 30 years (1981-2010) from the EC-EARTH and KNMI-RACMO22E RCP4.5 dataset for historic climate.

iLand uses climate data at the grain of resource units (100m resolution) and thus the climate data on 1km resolution was interpolated to 100m resolution using kriging methods (*gstat* package in R) with elevation as a variable. Digital elevation model (25m resolution) was obtained from EU-DEM v1.0 (http://land.copernicus.eu/pan-european/satellite-derived-products/eu-dem/eu-dem-v1-0-and-derived-products/view) and extracted for the study area. The mean elevation for each resource unit was determined from the DEM with zonal statistics tool in ArcMap. 30 year averages of historic climate dataset were used as a reference for the kriging. Variogram (*fit.variogram* in *gstat* package R) function was let to freely decide the best fitting variogram model and then apply it for each of the climate variables separately. After fitting the variogram with the climate data, the model was used to interpolate the values on resource unit grid based on the DEM values.

To reduce the database size, resource units were clustered based on the similarity between the climate variables using *clara* (*cluster* package) function in R. Input for the clustering were monthly long-term (1981-2010) means for all the climate variables (Fig. S6). The sensitivity of the climate variables to the number of clusters was analyzed and 150 clusters was found to be the best option producing at most ±4% differences between kriging and clustering values. Number of clusters beyond 150 did not significantly improve the results.


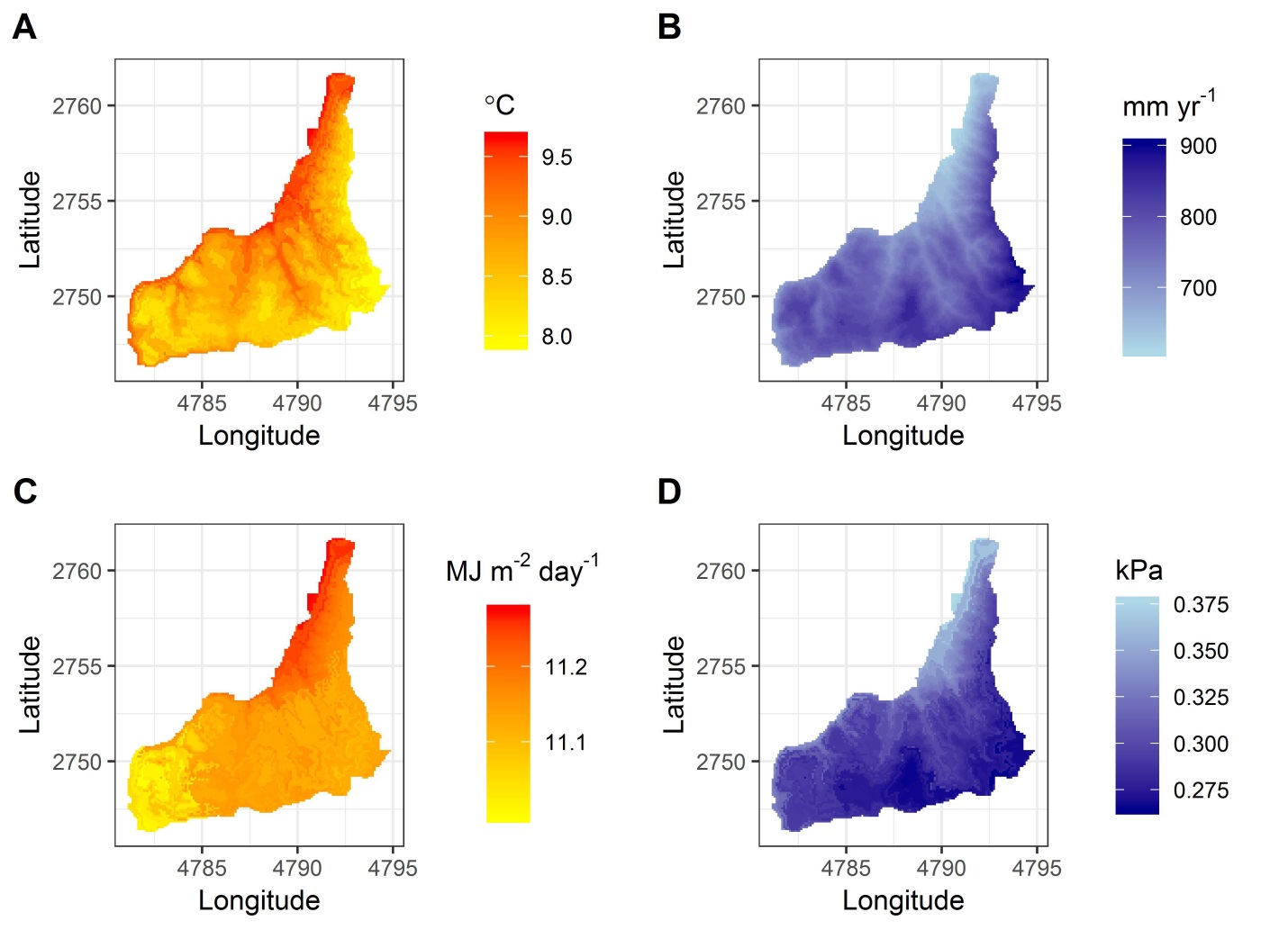


**Fig. S6.** Spatial variation of the kriged and clustered climate variables for the study landscape. Annual mean values for the historic climate dataset from 1981-2010: (A) temperature, (B) precipitation, (D) radiation and (E) vapour-pressure deficit.

Final step was to adjust the daily values from 1km grid data to the clusters. This was done by calculating the differences for climate variables between each cluster centroid resource unit and the 1km in which the centroid resource unit was located. The difference was absolute for temperatures and relative differences for *prec*, *rad* and *vpd*. Finally, daily values were adjusted for each cluster based on those differences by adding/subtracting temperatures, and multiplying *precip*/*rad*/*vpd* with the calculated differences. After doing the above procedure with the reference dataset, the final step of adjusting the cluster differences and the initial data was done for all of the climate change scenarios. The same cluster structure was used in all climate change scenarios.

The daily climate values were then stored for each climate scenario in their own SQLite database where each cluster with adjusted daily values appeared as their own table tagged with the cluster name (climate1,climate2,…,climate150). Historic climate for 2010-2100 was created by randomly resampling the years from 1981-2010 from scenario ICHEC RCP4.5. Climate data for all scenarios for time period 2101-2200 was randomly resampled from each scenario time period 2080-2100 (Fig. S7).


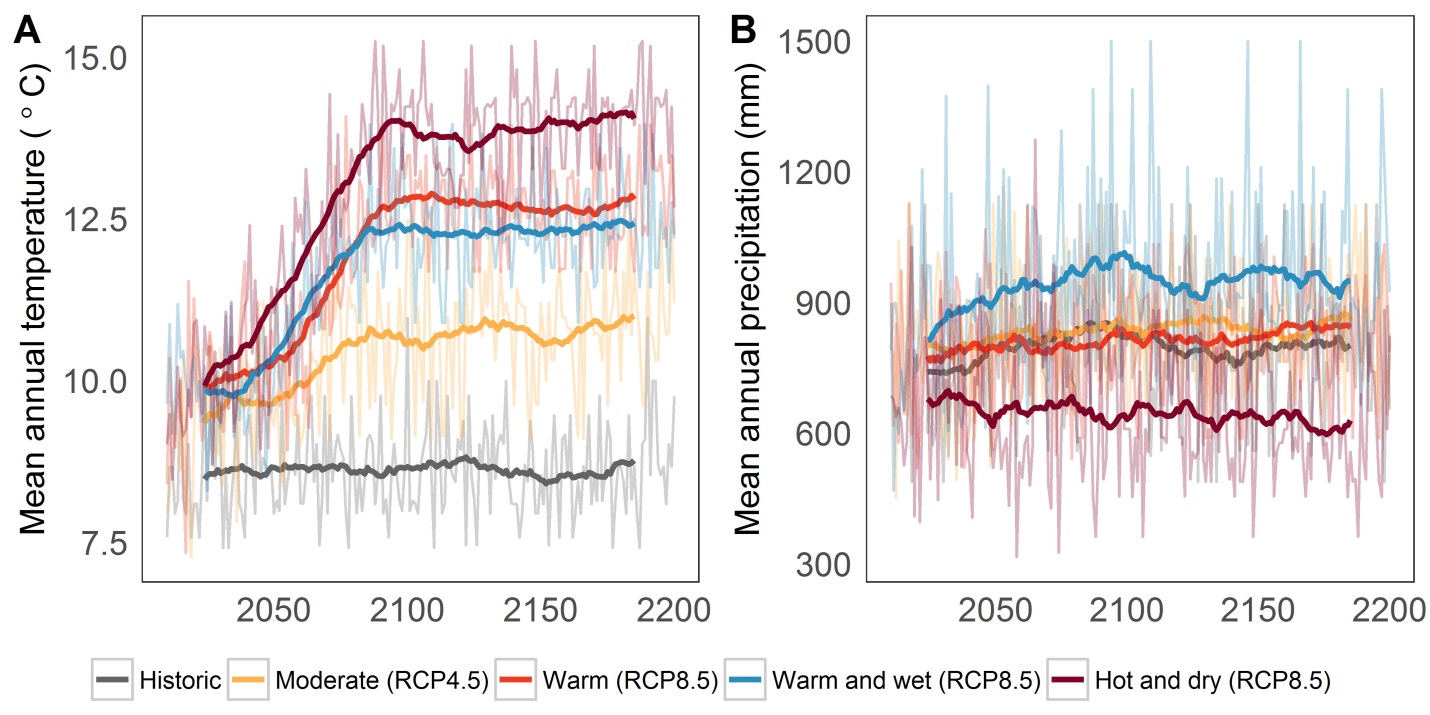


**Fig. S7.** Variation of mean annual temperature and precipitation in the study landscape over the whole study period from 2010-2200. The light lines are the interannual changes in the variables and the dark lines are 30 year running averages (±15 years)

**Supplementary Material S5: Soil data and carbon pools**

Soil data was obtained from the HydroBodNÖ project (Eder et al., 2011) as 50m resolution raster for multiple variables (sand, silt and clay share, soil depth, available nitrogen). The soil data is needed in the resource unit level (100m resolution) and thus was downscaled in R by calculating a mean value for each resource unit in the study landscape. These values were then used in iLand as input for the evaluation tests.

iLand needs deadwood, litter, and soil organic matter (SOM) pools as input carbon pools. Rasters for carbon pools of litter and SOM throughout the landscape were obtained by a stratified sampling from a soil carbon database (Seidl et al., 2009) using ecoregion and elevation as covariates. The amount of lying deadwood was assumed to be homogenous throughout the landscape and was estimated based on standing deadwood volume per hectare in Lower Austria based on Austrian Forest Inventory (6.7 m3 ha-1) by multiplying that with an estimate for standing:lying deadwood ratio for the area (1.71). This volume was then multiplied with the deadwood density (Paletto and Tosi, 2010) and finally multiplied with average carbon share in trees (0.5).

**Supplementary material S6. Resilience baseline values**

**Table S3.** Mean Norway spruce growing stock under historic climate used as a baseline value for the resielience metrics

|  | **Configuration** | | |
| --- | --- | --- | --- |
|  | **Dispersed** | **Clumped** | **Aggregated** |
| **Composition** | Norway spruce growing stock under historic climate, m^3^ ha^-1^ (resilience baseline) | | |
| 10% | 49.9 | 53.5 | 71.8 |
| 20% | 99.8 | 88.3 | 124.9 |
| 30% | 149.8 | 142.2 | 174.1 |
| 40% | 201.3 | 197.6 | 224.6 |
| 50% | 251.4 | 236 | 270.4 |

**Supplementary material S7. Natural disturbances results**

**Table S4.** Annual volume of Norway spruce timber disturbed by wind and bark beetles on average over the whole 190 year simulation period in different spatial configuration – species composition scenarios and under all climate change scenarios.

|  |  | **Species composition** | | | | | | | | | |
| --- | --- | --- | --- | --- | --- | --- | --- | --- | --- | --- | --- |
| **Climate scenario** | **Spatial configuration** | 10% | | 20% | | 30% | | 40% | | 50% | |
|  |  | *Wind*  *(m^3^ yr^-1^ ha^-1^)* | *Bark beetles*  *(m^3^ yr^-1^ ha^-1^)* | *Wind*  *(m^3^ yr^-1^ ha^-1^)* | *Bark beetles*  *(m^3^ yr^-1^ ha^-1^)* | *Wind*  *(m^3^ yr^-1^ ha^-1^)* | *Bark beetles*  *(m^3^ yr^-1^ ha^-1^)* | *Wind*  *(m^3^ yr^-1^ ha^-1^)* | *Bark beetles*  *(m^3^ yr^-1^ ha^-1^)* | *Wind*  *(m^3^ yr^-1^ ha^-1^)* | *Bark beetles*  *(m^3^ yr^-1^ ha^-1^)* |
|  | Dispersed | 0.27 | 0.06 | 0.37 | 0.17 | 0.45 | 0.3 | 0.57 | 0.46 | 0.77 | 0.64 |
| Historic | Clumped | 0.36 | 0.05 | 0.54 | 0.13 | 0.81 | 0.26 | 1.06 | 0.43 | 1.21 | 0.55 |
|  | Aggregated | 0.36 | 0.22 | 0.61 | 0.41 | 0.88 | 0.58 | 1.11 | 0.75 | 1.37 | 0.95 |
| Moderate | Dispersed | 0.28 | 0.12 | 0.38 | 0.38 | 0.47 | 0.68 | 0.59 | 1 | 0.78 | 1.33 |
|  | Clumped | 0.38 | 0.1 | 0.56 | 0.23 | 0.83 | 0.51 | 1.09 | 0.85 | 1.25 | 1.11 |
|  | Aggregated | 0.45 | 0.4 | 0.76 | 0.8 | 1.07 | 1.2 | 1.36 | 1.62 | 1.67 | 2.01 |
| Warm | Dispersed | 0.19 | 0.14 | 0.24 | 0.49 | 0.28 | 0.87 | 0.34 | 1.25 | 0.44 | 1.65 |
|  | Clumped | 0.29 | 0.12 | 0.4 | 0.3 | 0.58 | 0.62 | 0.74 | 1.04 | 0.83 | 1.36 |
|  | Aggregated | 0.34 | 0.45 | 0.55 | 0.9 | 0.75 | 1.34 | 0.94 | 1.78 | 1.14 | 2.24 |
| Warm and wet | Dispersed | 0.6 | 0.35 | 0.84 | 1.04 | 1.02 | 1.68 | 1.19 | 2.26 | 1.41 | 2.75 |
|  | Clumped | 0.56 | 0.17 | 0.8 | 0.41 | 1.15 | 0.84 | 1.48 | 1.38 | 1.68 | 1.75 |
|  | Aggregated | 0.6 | 0.49 | 1.03 | 0.96 | 1.41 | 1.42 | 1.8 | 1.91 | 2.2 | 2.37 |
| Hot and dry | Dispersed | 0.22 | 0.27 | 0.24 | 0.72 | 0.26 | 1.15 | 0.29 | 1.6 | 0.36 | 2.03 |
|  | Clumped | 0.23 | 0.16 | 0.3 | 0.36 | 0.39 | 0.69 | 0.48 | 1.07 | 0.53 | 1.34 |
|  | Aggregated | 0.24 | 0.38 | 0.34 | 0.75 | 0.45 | 1.1 | 0.55 | 1.46 | 0.65 | 1.84 |

**Supplementary material S8. Norway spruce recovery and impact**

**
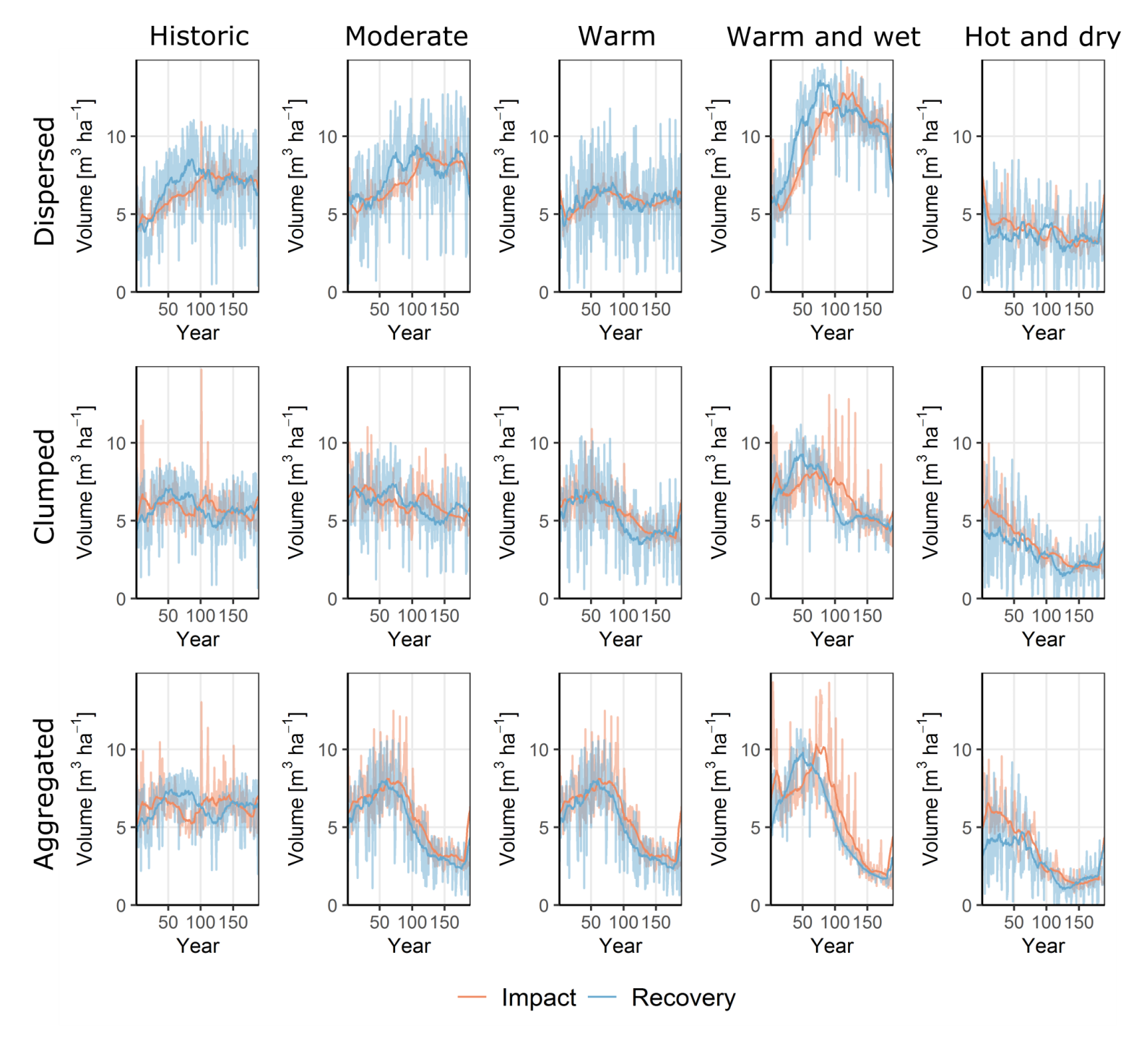
**

**Fig. S8** Temporal development of impact and recovery for a Norway spruce composition of 30% in the landscape with all spatial configuration and climate change scenarios. The faded lines represent the annual values of impact and recovery whereas the bolded lines are a 20 year running average (±10 year) indicating long term trends in the variables.

**Table S5.** Relative recovery and impact of climate change on Norway spruce in different landscape structure as well as climate change scenarios.

|  |  | **Composition** (% Norway spruce share) | | | | | | | | | |
| --- | --- | --- | --- | --- | --- | --- | --- | --- | --- | --- | --- |
| **Climate scenario** | **Configuration** | 10% | | 20% | | 30% | | 40% | | 50% | |
|  |  | *RelRecovery (% yr^-1^)* | *RelImpact (% yr^-1^)* | *RelRecovery (% yr^-1^)* | *RelImpact (% yr^-1^)* | *RelRecovery (% yr^-1^)* | *RelImpact (% yr^-1^)* | *RelRecovery (% yr^-1^)* | *RelImpact (% yr^-1^)* | *RelRecovery (% yr^-1^)* | *RelImpact (% yr^-1^)* |
|  | Dispersed | 3.91 | 3.70 | 3.89 | 3.73 | 3.88 | 3.75 | 3.89 | 3.79 | 3.94 | 3.89 |
| Historic | Clumped | 4.08 | 4.12 | 4.08 | 4.13 | 4.10 | 4.14 | 4.08 | 4.11 | 4.09 | 4.11 |
|  | Aggregated | 4.29 | 4.33 | 4.28 | 4.29 | 4.25 | 4.26 | 4.22 | 4.22 | 4.22 | 4.22 |
| Moderate | Dispersed | 4.46 | 4.12 | 4.44 | 4.16 | 4.37 | 4.15 | 4.26 | 4.11 | 4.19 | 4.14 |
|  | Clumped | 4.36 | 4.35 | 4.31 | 4.33 | 4.27 | 4.32 | 4.21 | 4.27 | 4.19 | 4.27 |
|  | Aggregated | 4.41 | 4.53 | 4.42 | 4.53 | 4.36 | 4.48 | 4.30 | 4.43 | 4.28 | 4.42 |
| Warm | Dispersed | 3.47 | 3.30 | 3.46 | 3.36 | 3.38 | 3.35 | 3.31 | 3.34 | 3.26 | 3.38 |
|  | Clumped | 3.80 | 3.91 | 3.71 | 3.86 | 3.64 | 3.82 | 3.53 | 3.73 | 3.48 | 3.71 |
|  | Aggregated | 3.59 | 3.93 | 3.49 | 3.82 | 3.36 | 3.71 | 3.34 | 3.69 | 3.32 | 3.67 |
| Warm and wet | Dispersed | 6.91 | 6.22 | 6.56 | 6.05 | 6.03 | 5.68 | 5.48 | 5.28 | 5.00 | 4.96 |
|  | Clumped | 5.02 | 5.03 | 4.80 | 4.88 | 4.56 | 4.69 | 4.37 | 4.53 | 4.26 | 4.45 |
|  | Aggregated | 3.81 | 4.22 | 3.69 | 4.11 | 3.60 | 4.02 | 3.56 | 3.98 | 3.54 | 3.96 |
| Hot and dry | Dispersed | 2.27 | 2.36 | 2.11 | 2.28 | 2.02 | 2.24 | 1.96 | 2.23 | 1.93 | 2.25 |
|  | Clumped | 2.23 | 2.59 | 2.12 | 2.50 | 2.04 | 2.43 | 1.97 | 2.36 | 1.92 | 2.32 |
|  | Aggregated | 1.92 | 2.37 | 1.88 | 2.31 | 1.85 | 2.28 | 1.82 | 2.25 | 1.83 | 2.27 |


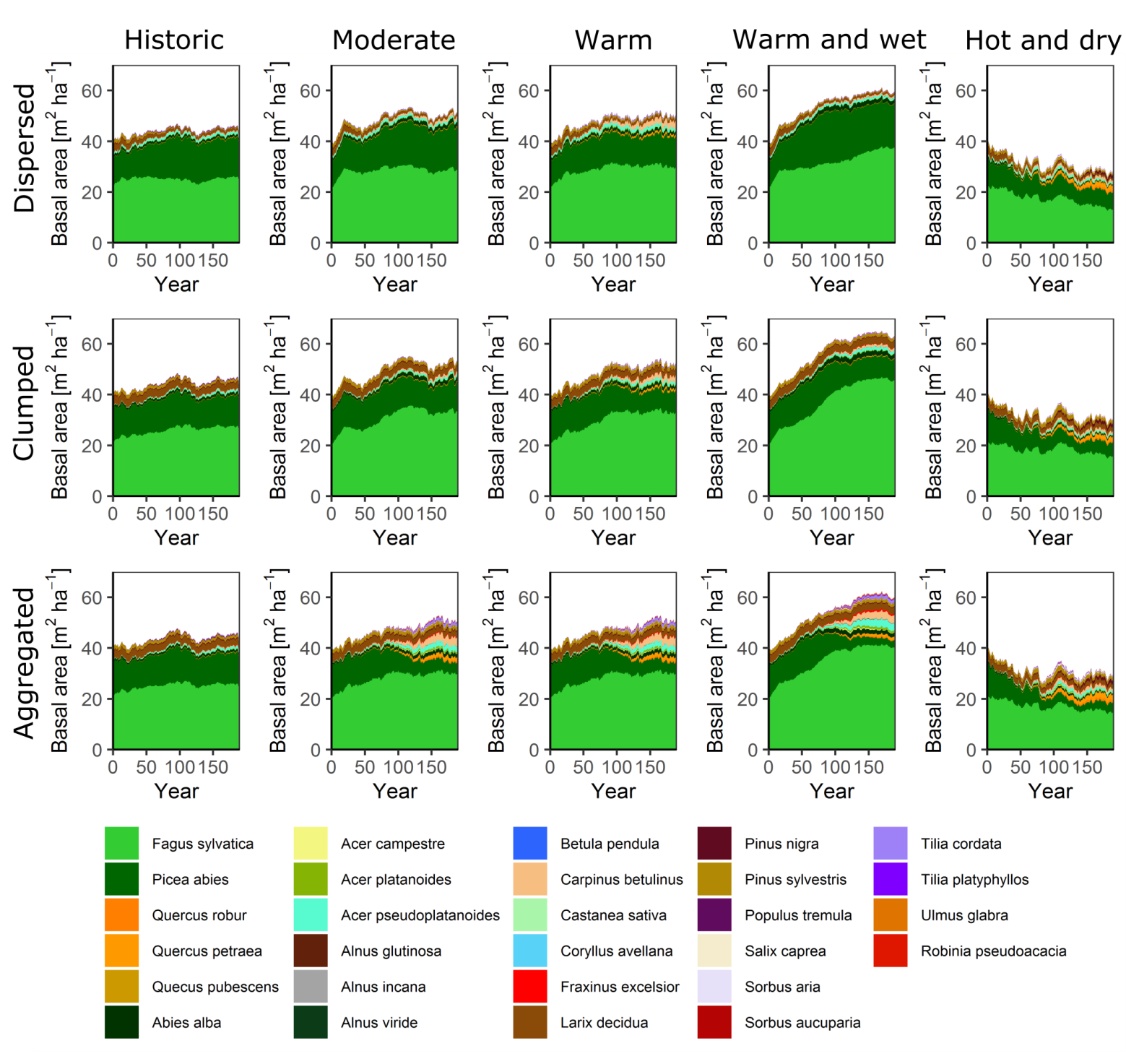


**Fig. S9.** The temporal species composition (i.e. mean basal area m^2^ ha^-1^) changes with a 30% Norway spruce share under different landscape configuration and climate change scenarios

**References:**

Eder, A., Sotier, B., Klebinder, K., Sturmlechner, R., Dorner, J., Markart, G., Schmid, G., Strauss, P., 2011. Hydrologische Bodenkenndaten der Böden Niederösterreichs (HydroBodNÖ).

Haiden, T., Kann, A., Wittmann, C., Pistotnik, G., Bica, B., Gruber, C., Haiden, T., Kann, A., Wittmann, C., Pistotnik, G., Bica, B., Gruber, C., 2011. The Integrated Nowcasting through Comprehensive Analysis (INCA) System and Its Validation over the Eastern Alpine Region. Weather Forecast. 26, 166–183. doi:10.1175/2010WAF2222451.1

Lexer, M.J., 2001. Simulation der potentiellen natürlichen Vegetation für Österreichs Wälder. Vergleich von statischen und dynamischen Modellkonzepten (No. Band 16), Forstliche Schriftenreihe.

Paletto, A., Tosi, V., 2010. Deadwood density variation with decay class in seven tree species of the Italian Alps. Scand. J. For. Res. 25, 164–173. doi:10.1080/02827581003730773

Pretzsch, H., Biber, P., Schütze, G., Uhl, E., Rötzer, T., 2014. Forest stand growth dynamics in Central Europe have accelerated since 1870. Nat. Commun. 5, 1–10. doi:10.1038/ncomms5967

Seidl, R., Rammer, W., Lexer, M., 2009. Schätzung von Bodenmerkmalen und Modellparametern für die Waldökosystemsimulation auf Basis einer Großrauminventur. Allg. Forst-Jagdztg 180, 35–44.

Thom, D., Rammer, W., Garstenauer, R., Seidl, R., 2018. Legacies of past land use have a stronger effect on forest carbon exchange than future climate change in a temperate forest landscape. Biogeosciences 15, 5699–5713. doi:10.5194/bg-15-5699-2018
